# Mobile Technologies and Geographic Information Systems to Improve Health Care Systems: A Literature Review

**DOI:** 10.2196/mhealth.3216

**Published:** 2014-05-08

**Authors:** José António Nhavoto, Åke Grönlund

**Affiliations:** ^1^InformaticsÖrebro University School of BusinessÖrebro UniversityÖrebroSweden

**Keywords:** health care, eHealth, mobile technology, mobile phone, SMS, text messaging, geographic information system, GIS

## Abstract

**Background:**

A growing body of research has employed mobile technologies and geographic information systems (GIS) for enhancing health care and health information systems, but there is yet a lack of studies of how these two types of systems are integrated together into the information infrastructure of an organization so as to provide a basis for data analysis and decision support. Integration of data and technical systems across the organization is necessary for efficient large-scale implementation.

**Objective:**

The aim of this paper is to identify how mobile technologies and GIS applications have been used, independently as well as in combination, for improving health care.

**Methods:**

The electronic databases PubMed, BioMed Central, Wiley Online Library, Scopus, Science Direct, and Web of Science were searched to retrieve English language articles published in international academic journals after 2005. Only articles addressing the use of mobile or GIS technologies and that met a prespecified keyword strategy were selected for review.

**Results:**

A total of 271 articles were selected, among which 220 concerned mobile technologies and 51 GIS. Most articles concern developed countries (198/271, 73.1%), and in particular the United States (81/271, 29.9%), United Kingdom (31/271, 11.4%), and Canada (14/271, 5.2%). Applications of mobile technologies can be categorized by six themes: treatment and disease management, data collection and disease surveillance, health support systems, health promotion and disease prevention, communication between patients and health care providers or among providers, and medical education. GIS applications can be categorized by four themes: disease surveillance, health support systems, health promotion and disease prevention, and communication to or between health care providers. Mobile applications typically focus on using text messaging (short message service, SMS) for communication between patients and health care providers, most prominently reminders and advice to patients. These applications generally have modest benefits and may be appropriate for implementation. Integration of health data using GIS technology also exhibit modest benefits such as improved understanding of the interplay of psychological, social, environmental, area-level, and sociodemographic influences on physical activity. The studies evaluated showed promising results in helping patients treating different illnesses and managing their condition effectively. However, most studies use small sample sizes and short intervention periods, which means limited clinical or statistical significance.

**Conclusions:**

A vast majority of the papers report positive results, including retention rate, benefits for patients, and economic gains for the health care provider. However, implementation issues are little discussed, which means the reasons for the scarcity of large-scale implementations, which might be expected given the overwhelmingly positive results, are yet unclear. There is also little combination between GIS and mobile technologies. In order for health care processes to be effective they must integrate different kinds of existing technologies and data. Further research and development is necessary to provide integration and better understand implementation issues.

## Introduction

The proliferation of mobile phones has provided a powerful communication channel to strengthen health information systems. Functional and structural properties of mobile phones, such as low start-up cost, text messaging, and flexible payment plans, make them attractive to use for contacts with patients in various health care processes. Often they are used to disseminate information to patients, but when used in conjunction with health care–related software apps, they can also provide real-time feedback needed to monitor treatment compliance or effect, and also serve as data collection tools. Further, back-end systems connected to mobile phones have the capability to serve as a platform for enabling preprogrammed, portable, automated services, which can make health care, and health information systems, increasingly decentralized.

Today, there is a lot of effort put into using mobile communication to improve various processes in health care, preventive as well as reactive. This is done in many ways, for example, by keeping doctors and patient better in touch (eg, by reminder systems), by keeping local health care centers in better touch with central hospitals (eg, by local doctors sending images for expert analysis), but also by providing preventive health information so as to decrease the number of people who become patients (eg, by support in leading a more healthy life), and providing better statistics so as to better plan actions and resource allocation in the health care system, such as, in conjunction with natural disasters or epidemics.

Many, if not all, of the systems used for the above purposes require backend systems, or will at least perform better if they have such. For instance, reminders to patients about visiting the doctor or taking their medicine must be integrated with a patient record so as to avoid a huge amount of manual labor. A text messaging (short message service, SMS) system for reporting cases of HIV/AIDS at a local health care center to a central hospital should be integrated with some apps for producing statistics and informing relevant actors about the development; for example, the developments of the number and the nature of cases at different care centers might vary over time that may require redistribution of resources so as to provide effective care. More generally, to be effective with respect to all stakeholders in health care, data collection systems should be technically integrated with systems for communication and decision making. As there is much health care data around, and many variables involved in making good decisions, medical as well as administrative, spatial, and economic, there is a need for effective data handling, analysis, and presentation. For instance, health data from various regions in a country could be presented in geographic information systems (GIS) so as to provide better means of communication to decision makers. It may make it easier to understand data by using graphical presentation, and it may make it easier to analyze data as they can be coupled with other data (eg, regarding population, geography, and economy), which may distinguish different regions from each other. Taking all such factors into consideration may be necessary for the purpose of optimizing the allocation of available health care resources across a country and making sure effective methods are used everywhere.

This research, therefore, looks into both technologies employed in operative processes of health care, the mobile phones, and technology aimed at providing support for decisions, the GIS, within health care. The study searches for cases where the two types of technologies are integrated and, based on the assumption that the integration would generally be as low as technology is relatively new, for clues to how best do this integration; what are the needs and the potential gains?

The purpose of this article is to provide a review of literature related to the use of mobile technologies and GIS in health-related research for improving health care. The major topics for the review are the use of mobile technologies and GIS to improve health care. The research questions that served as the basis of this literature review are:

1. What is the geographical distribution of publications on mobile technologies and GIS?
2. How have mobile technologies and GIS been used to improve health care?
3. What were the effects associated with the use of mobile technologies and GIS?

## Methods

### Search Criteria

This is a literature study aiming at identifying the state of the art in mHealth, use of mobile phones for communication with patients, and GIS as well as research gaps. A thorough search in prominent databases was conducted using predetermined keywords. The search targeted articles written in English and published in 2005 or later. The year 2005 was chosen because the literature on interventions using mobile technologies has increased substantially over the past few years, and apps before 2005 are not only rare, but would also be expected to be tentative in nature as mobile technology was generally less mature at that time, as concerns end user units as well as networks. Papers found were screened for relevance (ie, to confirm that they reported the use of mobile technologies or of GIS, leaving a resulting set of papers for full-text eligibility assessment.

### Search Strategy

Articles were systematically identified through a combination of computerized database searches and manual searches of the reference lists in relevant articles found. The databases PubMed, BioMed Central, Scopus, ScienceDirect, and Web of Science were used. The search was restricted to studies reported in English-language journals and indexed with the following keywords: cell phone, mobile phone, SMS, text message combined with health; and GIS combined with health. The search was restricted to title and abstract fields, to avoid retrieving articles, which were not focusing on these things yet mentioned the terms.

### Exclusion/Inclusion Criteria

The following criteria were used for inclusion/exclusion of articles: (1) the literature review concentrates on research published from 2005 to 2012 (the first search was in December 2012 and the last in April 2013), (2) the study excluded research published before 2005 and also excluded non-English language publications, (3) articles had to be published in peer-reviewed journals and conference proceedings, and (4) articles addressing the use of mobile technologies had to use uni/bidirectional communication. Further, we only included articles where data could be extracted or, at a minimum, where the abstract was available.

## Results

### Search Results

**Figure 1:**
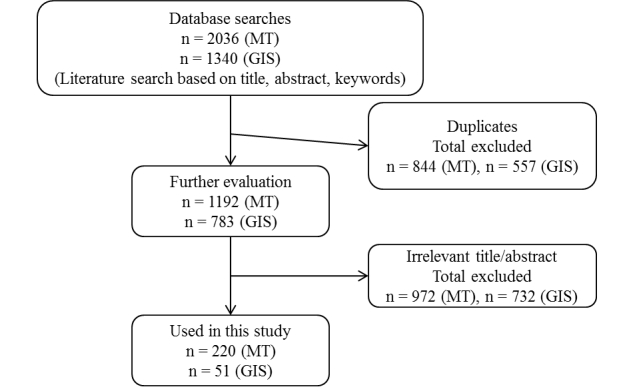
Article selection process.

The initial combined database search yielded 3376 articles (Figure 1). A title and abstract review was conducted, from which we identified 271 articles that met the eligibility criteria.

Given the large sample size, we further analyzed these articles and organized them into the following categories: mobile technologies: (1) treatment and disease management (n=34), (2) data collection and disease surveillance (n=29), (3) health support systems (n=38), (4) health promotion and disease prevention (n=50), (5) communication to or between health care providers (n=60), and (6) medical education (n=9). GIS: (1) disease surveillance (n=12), (2) health support systems (n=12), (3) health promotion and disease prevention (n=19), and (4) communication to or between health care providers (n=8). Of note, some papers sometimes overlapped in different categories, but we categorized them based on the technology use’s primary purpose. For example, if the technology was aiding patient care via telediagnosis, then we placed the article in the category of communication.

### Geographical Distribution of Publications

In terms of geographical spread of publications, Table 1 indicates that the highest number of publications were from developed countries.

**Table 1:** Geographical distribution of publications between 2005 and 2012.

Country	Number of papers
Angola	1
Australia	8
Austria	4
Brazil	3
Cameroon	1
Canada	14
China	7
Congo	1
Croatia	1
Denmark	2
France	5
Germany	1
Ghana	1
Greece	1
India	10
Iran	1
Ireland	5
Israel	4
Italy	3
Japan	4
Kenya	12
Korea	11
Malaysia	3
Mexico	1
New Zealand	7
Nicaragua	1
Norway	5
Peru	9
Saudi Arabia	2
Singapore	2
South Africa	3
Spain	2
Swaziland	1
Sweden	2
Taiwan	2
Tanzania	2
Thailand	2
the Netherlands	6
Trinidad and Tobago	2
Uganda	3
UK	31
USA	81
Vanuatu	3
Zambia	1
Total: 44 countries	271 papers

### Research Findings by Category

#### Treatment and Disease Management

Interventions related to treatment and management focused on investigating patient adherence to treatment (eg, visiting a doctor as planned) [1-6], adherence to medication [7-13], and disease management (particularly for diabetes [14-23] and asthma [24-26]), including coordinated health care interventions between health care providers and patients using communication technologies (mobile phone-based apps and SMS) for patients self-care of chronic diseases. The literature contains 28 cases with sample sizes ranging from 25 to 424. The interventions are described in Multimedia Appendix 1. Interventions in both developed countries (United States [1,16,17,22-24,27-31], Ireland [2,11], United Kingdom [5,25,26], Denmark [6], Spain [9], France [10], the Netherlands [12,13], South Korea [14,18,32], Austria [19], and Canada [20,33]), and developing ones (Peru [7,8], Kenya [3,34], Cameroon [35], and Brazil [36]) have explored the use of mobile phone-based software, voice and SMS, and personal digital assistants (PDAs). In these interventions, the technology was used to send automated reminders to patients, either by voice or text messages. In addition to reminders from health care units to patients, health care units may receive text message from patients.

In interventions regarding adherence to treatment and to medication, mobile technologies were used to send reminders so as to improve antiretroviral therapy (ART) in HIV-infected adolescents [1]; send at least one medication-specific dosage reminder for a chronic oral medication [31]; measure asthma medication use and symptoms [30]; and to reduce out-patient clinic nonattendance [5]. Reminders were sent 3 days prior to patients’ out-patient clinic appointment [2]; to HIV-positive patients with support content and enquire into how they are doing [3]; to patients to take their anti-asthmatic medication [6]; to soldiers to take their malaria chemoprophylaxis [10]; to patients to take antidepressant medication [11]; to patients with type 2 diabetes to take their oral antidiabetics [12,13]; to patients to take their oral antipsychotic medication [9]; and to take medications and provide additional support [34]. In addition, reminders were sent with instructions to patients reminding them to apply their morning and evening topical acne medication [28]; to support antiretroviral medication adherence [7,8]. Thus, motivational text message were sent to HIV-positive adults for adherence to ART [35]. In some interventions, patients received reminders 30 minutes before patients’ last scheduled time for a dose of medicine during the day [36]; received reminders and were asked to acknowledge receiving their messages after taking the vitamins [33], and were required to send event-based messages whenever they experienced asthma symptoms or took asthma rescue or controller medications, and they received time-based messages daily that prompted for a response about asthma medications or symptoms [37].

For disease management, interventions focused on the use of mobile phone-based apps and SMS. Interventions using mobile phone-based apps used the technology to transmit blood glucose levels using a mobile phone with a glucometer integrated into the battery pack on management of type 2 diabetes to the Internet-based glucose monitoring system [14]; transmit blood glucose meter readings using a mobile phone with a glucometer integrated to the Internet-based glucose monitoring system [22]; transmit blood glucose levels to secure servers and receive real-time feedback [27]; and transmit blood glucose values, diabetes medications, and lifestyle behaviors to a remote server and receive real-time educational and behavioral messaging [23]. The same apps are also used to transmit peak flow reading and symptom score to secure server with immediate feedback of control and reminder of appropriate actions in supporting asthma self-management [25,26], and transmit diabetes-related data with synchronization to the remote database at the monitoring center [19]. The SMS function was to send personalized medication, and appointment reminders and text messages were received from patients on adherence [16]; send tailored daily messages prompting patients with type 2 diabetes to enhance their diabetic self-care behavior [17]; send peak flow reading each day to a Web server and receive a text reminder if they did not send it by 11 AM [24].

In these interventions mobile phone-based apps and SMS were found to be acceptable to patients [11,16], practical and acceptable [1], feasible [10,16,17,24], effective [9,14], and cost-effective [5,19,38]. Patients had positive perceptions [6,8,15,19-21,25], positive impact on some clinical outcomes (eg, medication taking) [17,18,23,26,27], and were highly satisfied [32]. These apps and SMS improved patient adherence to medication [12,18,31,34], and to health behavior (taking vitamin C for preventive reasons) over a 1-month intervention [33]; and they also assisted in preserving higher rates of adherence over time [31]. In addition, their use increased adherence of HIV-infected women to ART drug-based treatment regimen [36], increased adherence to asthma-preventer inhaler [37], and significantly improved self-reported adherence to ART [29]. In particular, SMS reminders are a simple and cost-effective way to improve nonattendance [2]; and provided an ubiquitous, easy-to-use, and cost-efficient solution to assist diabetes patients on intensive insulin treatment [19].

SMS were not associated with significant differences in adherence to topical medications in patients with mild to moderate acne and had no significant effect on therapeutic response [28]. They also did not significantly improve adherence to ART [35].

In most cases, in the above literature, the mobile phones were used to send messages from a health care center to patients, most commonly reminders for taking medicine, visiting the doctor, etc. In some cases there was integrated data collection, for example, by integrating a glucometer into the battery pack on management of type 2 diabetes or transmitting data on asthma symptoms.

In 11 cases, the front-end mobile system was connected to some back-end system. Most commonly this was done by sending automated reminders based on some database (eg, with data collected from patient records) but in some cases also incoming data (from patients) were automatically inserted in a database, such as, into an Internet-based glucose monitoring system.

#### Data Collection, Reporting, and Disease Surveillance

On this topic the survey found 28 studies using mobile communication technologies with samples ranging from 8 to 648 participants, and eight studies using GIS technologies. The interventions are described in Multimedia Appendices 2 and 3. Interventions using mobile communication technologies in both developed countries (Sweden [39], Norway [40,41], USA [42-45], Germany [46], Canada [47,48], UK [49,50], Austria [51], Japan [52,53]), and developing ones (Ghana [54], Kenya [55-57], Peru [58-60], Angola [61], Swaziland [62], Malaysia [63], India [64,65], Iran [66], China [67]) have investigated the use of mobile phone-based apps, mobile phone functions (SMS and voice), and PDAs. In these interventions, the technology was used to collect or report health data (eg, influenza vaccination, tuberculosis, and HIV) and for disease surveillance (eg, tracking infectious disease, communicable disease, and respiratory infections).

For interventions using the SMS and or voice functions of mobile phone, one [54] evaluated the acceptability of using SMS for reporting postpartum hemorrhage data; one [39] compared the feasibility of using SMS and telephone in collecting self-reported data about influenza vaccination; two [62,63] evaluated the effectiveness of using SMS, the first for delivering laboratory results and the second for patients’ weekly symptoms reports. In addition, one [45] investigated the feasibility and acceptability of using two-way SMS texts communication to collect situational assessment data; one [64] provided a quantitative evaluation of data entry accuracy using SMS when compared with Internet and voice; one [65] investigated the effectiveness and efficiency gains in using mobile apps for detecting disease outbreaks in near-real-time; and one [49] tested the reliability, validity, acceptability, and practicability of SMS messaging for collecting patients’ infant feeding method and future feeding plans.

For interventions using PDAs, two studies [40,55] evaluated acceptability, two [40,55] data quality, one [55] usefulness, one [59] efficiency, three [46,56,60] effectiveness of a PDA-based system compared with paper-based system; one [40] assessed how PDAs performed as collection tools of patient-reported outcomes in clinical research compared to pen and paper diaries in terms of feasibility, protocol compliance, data accuracy, and subject acceptability. In addition, one [41] compared daily and weekly registrations of self-reported health status measures between PDA and paper-pencil (PP) format regarding scores, variation, and feasibility; one [42] feasibility of using PDA-based system for tracking and analysis of food intake for pregnant women; one [43] compared the completeness of data collection using a paper and PDA-based system. Other studies, assessed feasibility and patient acceptance of PDAs for collection of health data [68]; one [58] evaluated the quality of data on sexual behavior data collected with PDA-based system in comparison with paper-based questionnaires; and one [61] explored the acceptability of PDA for HIV/AIDS data collection and to identified potential barriers to acceptance.

For interventions using PDAs the technology was used to send individual’s usual food intakes to registered dieticians for analysis [52,53]. Some benefits of using PDA-based system or mobile phones (voice and/or SMS) include improved data quality [56,65], improved data completeness [43,56], reduction in staff work hours [59,60], led to reduction in processing time [60], reduced errors in data entry [60], positive feedback from users [41,58], and lower number of inconsistencies and missing values [58,62]. In addition, some studies have adopted the use of open-source tools, which contributes to cost effectiveness [58,60]. Mobile phones were also found to be a useful tool for communication in conjunction with infectious disease surveillance in areas hit by natural disasters [67].

Disease surveillance is a field where GIS technology has been much used. GIS for disease surveillance is an epidemiological practice that monitors the spread of disease in order to establish patterns of progression. Examples of diseases having been monitored in both developed countries (Canada [69], France [70], US [71,72], the Netherlands [73]), and developing ones (South Africa [74], Nicaragua [75], India [76], Vanuatu [77], Congo [78], Trinidad and Tobago [79]) using GIS include injury [74], respiratory and acute gastrointestinal illness [71], HIV/AIDS [72,73,76,78], malaria [77,80], rabies epidemic [79], dengue fever [75], West Nile virus [69], and communicable disease [70]. In these interventions, GIS contributed to assessing visit rates for common illnesses in a defined community and identified spatial variability over time [71]; provided an effective and efficient operational tool for rapidly defining spatial distribution of malaria [77]; provided a useful tool to track trends in HIV incidence, HIV prevalence, and related risk behavior in a vulnerable population of women [72]. In addition, GIS provided a tool for analyzing risk factors that increase HIV infections [78], to accurately identify areas with high incidences of mosquito infestation and interpret the spatial relationship of these areas with potential larval development sites, such as garbage piles and large pools of standing water [75]. GIS also facilitated the collection, localization, management, and analysis of monitoring data; it also allowed for the display of the results of analysis on maps, tables, and statistical diagrams [69]; and supported key elements of surveillance-response, including understanding epidemiological variation within target areas, implementing appropriate foci-specific targeted response, and consideration of logistical constraints and costs [80].

#### Health Support Systems

On this topic, the survey found 38 studies using mobile communication technologies with samples ranging from 5 to 5800, and 26 studies using GIS technologies. The interventions are described in Multimedia Appendices 4 and 5. Interventions using mobile communication technologies in both developed countries (Norway [81,82], France [83], UK [84-89], Spain [90], US [91-97], Sweden [98], Greece [99], the Netherlands [100], Italy [101], New Zealand [102-104], and South Korea [105]), and developing ones (Saudi Arabia [106], Tanzania [107,108], Peru [109-111], South Africa [112], Uganda [113], China [114,115], Trinidad and Tobago [116], Kenya [117], India [118]) have investigated the use of mobile phone-based apps (six studies); mobile phone functions (SMS, 20 studies), voice (three studies) and multimedia messaging service (MMS; two studies), and PDAs (10 studies).

Mobile phone-based apps were to design a mobile dietary management support system for people with diabetes [81], develop a smoking cessation support system [86], and support type 1 diabetes self-management [99]. These apps were also designed to support self-care process for patients with both diabetes and cardiovascular disease [116], and help individuals with weight problems to lose or maintain weight [93].

SMS-based technologies were used to integrate with an electronic medical record system on nonattendance rates in outpatient clinics [106]; support antimalarial stock management [107]; provide real-time collection and transmission of adverse events related with metronidazole administration for treatment of vaginosis among female sex workers [110]; provide tailored and stage-specific cessation messages to college smokers [94]; and provide on-going behavioral reinforcement for HIV-positive men who have sex with men [97]. In addition, the technology was used to develop a staff recall system for use after mass casualty incident [91] and to develop a support system to enhance self-efficacy, facilitate uptake of intensive insulin therapy and improve glycemic control in pediatric patients with type 1 diabetes [85]. The technology was also used to improve smoking cessation rates [87]; improve pain reporting, pain measurement, and adequate pain therapy for patients with cancer [100]; promote blood glucose monitoring [92]. Additionally, they were used to deliver tailored smoking cessation advice [88,96]; deliver individualized pharmaceutical care for medication compliance and safety [114]; deliver an automated support to young people with diabetes [89]; to collect, verify, and manage data on HIV/AIDS stigma and pregnancy in a rural area [117]; and transmit medical data collected and home by health care professionals [101].

PDA-based technologies were used to develop a PDA-based electronic system to collect, verify, and upload bacteriology data into an electronic medical record system [109]; develop a wireless clinical care management system [108]; and develop a data collection/entry system for public surveillance data collection [115]. In addition, they were used to display audio-visual animation for patient education in a clinical setting [84]; for medical professionals to access a centralized database system of patient data [105]; to assist tuberculosis-control programs to trace patients who interrupt treatment [112]; collect data from HIV patients and to support antiretroviral adherence [111]; and for dietary self-monitoring in patients with type 2 diabetes [95].

The use of mobile phone-based apps as health support systems doubled the self-reported quit rate in the short term [86] and improved patients’ self-management of type 1 diabetes mellitus [99]. The use of mobile phone functions (SMS, MMS, and voice) showed a positive effect on the reduction of the nonattended appointments [106]; provided visibility of antimalarial stock levels to support more efficient stock management [107]; was feasible, safe, did not alter quality of life, and was associated with a trend toward improved metabolic control [83]. The technologies also rapidly mobilized sufficient numbers of anesthesia personnel in response to a mass casualty incident [91]; improved self-efficacy and adherence to uptake of insulin therapy [85]; significantly improved smoking cessation rates [87]; provided patients with rapid, effective medication guidance and pharmaceutical care after discharge [114]; proved to be a productive channel of communication to promote behaviors in overweight adults [93], and was potentially efficacious and easily disseminated method for providing smoking cessation interventions to young adult smokers [94].

The use of PDA-based technologies as health support systems significantly decreased delays in processing and errors with a positive user experience [109]. They provided a fast and efficient data communication mechanism [105]; were a useful and user-friendly medical decision support system for nurses in home care [98]; showed promise as a tool for assisting those with type 2 diabetes in their efforts to manage their disease [95]; and reduced or eliminated data entry errors, performed better in timeliness of receipt and data handling that PP and provided a cost-effective alternative to the paper-based [115].

Interventions using GIS technology in both developed countries (Australia [119,120], UK [121], US [122,123], Canada [124,125], France [126]), and developing ones (Zambia [127], Vanuatu [77,128], Mexico [129], Saudi Arabia [130]) have used the technologies to identify optimal settings for cancer prevention and control [131]; deployment, monitoring, and evaluation of entomological interventions for malaria control [127]; assess environmental exposure [122]; share disease information [124]. Additionally, the GIS was used to support public health decision making for end-stage renal disease [126]; support malaria elimination [77,128]; estimate population catchment area around specific health services in rural and remote areas [125]; prevention and control of vector-borne diseases [129]; and evaluate the spatial distribution of hospital demand and for defining hospital service area [130].

The use of GIS has enabled detection of spatial trends of parasite prevalence following extensive deployment of front line vector control interventions; improved the tracking of entomological indicators: species characterization and insecticide resistance status, including falciparum malaria parasite prevalence and impact assessment of insecticide treated nets and indoor residual spraying (IRS) [127]. GIS was found to be a useful tool in displaying environmental risk factors and potentially associated health effects [122]; enabled cross-border visualization, analysis, and sharing of infectious disease information through interactive maps and/or animation in collaboration with multiple partners via a distributed network [124]; and provided an effective and efficient operational tool for rapidly defining the spatial distribution of target populations in designated malaria elimination zones [77]. In addition, it has empowered program managers at the provincial level to implement and asses the IRS intervention with the degree of detail required for malaria elimination [128]; and helped health planners on evaluating the spatial distribution of hospital demand and for defining hospital service area [130]. In special, using online analytical processing (OLAP)-GIS decision support system, tasks were completed more efficiently, with a higher rate of success, and with greater satisfaction [123].

#### Health Promotion and Disease Prevention

On this topic, the survey found 48 studies using mobile communication technologies with samples ranging from 5 to 5800, and 60 studies using GIS technologies. The interventions are described in Multimedia Appendices 6 and 7. Interventions using mobile communication technologies in both developed countries (US [132-154], Norway [155], Ireland [156,157], the Netherlands [158,159], Austria [160], New Zealand [161,162], Australia [163-165], and South Korea [166,167]), and developing ones (Kenya [168], Croatia [169], Singapore [170], Brazil [171], China [172-174], India [175-177], Malaysia [178,179]) have investigated the use of mobile phone-based apps, mobile phone voice call, SMS, and PDAs.

For interventions using mobile phone-based apps the technology was used to transmit patients’ self-reported outcomes [180]; transmit self-monitoring data to a website for review and analysis by clinicians, parents, and patients [134]; and ocate missing persons with dementia [145]. Patients were also able to transmit twice daily recording of symptoms, drug use, and peak flow with immediate feedback prompting action according to an agreed plan or paper-based monitoring [143].

For interventions using mobile phone functions the technologies were used to send SMS reminders to patients that enabled improving adherence to sunscreen app [132], and send immunization reminders to children aged 11 to 18 years that needed either or both meningococcal and tetanus-diphtheria-acellular pertussis immunizations [133]. Particularly, text messages were used to send result after genital chlamydia trachomatis infection tests [142]; send reminders about pediatric malaria case-management to health workers [168], and to women toward the end of her menstrual period to do breast self-examination [176]. In some cases, patients sent their peak expiratory flow results daily and received feedback from health center [169]; patients sent weekly self-monitoring data on exercise and eating behavior and their mood, and in return they received tailored feedback messages [158]; patients transmitted images of their psoriasis (chronic skin disease) lesions and received feedback message [160]; patients sent a weekly message regarding their bulimic symptomatology and mood states and received automatic feedback [146]; monitor symptoms in patients with asthma [170]; monitor the functional mobility of elderly subjects in an unsupervised environment [156]; transmit blood pressure measurement unit from patients to a remote server and send notification information to users if patient’s blood pressure is abnormal [173]; support educational intervention for patients with diabetes [166,167]; improve treatment results and reduce dropout rates in children with overweight [159]; remotely monitor the long-term mobility levels of elderly people in their natural environment [157]; monitor patients’ appetite ratings hourly over 7 consecutive days [135]; provide smoking cessation advice, support, and distractions for patients willing to quit smoking [161,162]; provide voice counseling sessions to HIV-positive population [137,138]; send tips on healthy eating and physical activity, as well as reminders to drink water and expressions of encouragement in a weight management program [139]; send sexual health education messages to young people aged between 16 and 29 years recruited from a music festival in Melbourne [163].

In particular, text messages were used as a tool to send reminders. Reminders were sent to promote receipt of influenza vaccination among children and adolescents [140]; to patients at pediatric clinic for appointments [149,151]; via text or call to patients after their missed clinic appointments [148,152]; via text or call to patients prior scheduled appointment [147,153,154,164,171,172,177-179]; and via text to patients after 7-days circumcision [141].

Some benefits of using mobile technologies as health support systems were improved attendance in return general ophthalmology clinic patients [147,150]; improved attendance rate in primary care [178]; improved attendance at the 7-day post-operative clinic visit following adult male circumcision [141]; improved immunization coverage in a low-income, urban population [133]; and improved and maintained health workers’ adherence to treatment guidelines for outpatient pediatric malaria [168]. In addition, the technologies have improved asthma control when added to a written action plan and standard follow-up [169]; improved attendance rates at a health promotion center [174]; improved levels of glycosylated hemoglobin and 2 hours post meal glucose in type 2 diabetes patients [166,167]; improved health knowledge and sexually transmitted infections testing [163]; and significantly improved follow-up adherence in pediatric cataract treatment [172]. Thus, the technologies reduced failure to attend rate at outpatient clinics [148,149,151,164,165,171]; reduced the number of failed appointments significantly [153,177]; offered a time-, labor-, and cost-efficient strategy for encouraging engagement with psychiatric outpatient services [154]; and offered an innovative, low-cost and effective method of improving adherence to sunscreen app [132]; saved staff time per month and reduced number of days to diagnosis [142].

In particular, SMS was cost-effective compared with voice call reminder [150,178]; was a cost-effective method for improving patient attendance at dental appointments [153]; reduced dropout rates from a pediatric lifestyle intervention [159]; reduced the financial burden on health care services by facilitating more efficient use of health care resources [157]; doubled quit rates at 6 weeks [162]; decreased symptoms of distress while increasing self-efficacy [138]; increased short-term self-reported quit rates [161]; increased rate of influenza vaccination [140]; increased the practice of breast self-examination [176]; and increased adherence of obese adolescents enrolled in a weight management program [136]. Routine SMS texting was a cost-effective means of reducing nonattendance rates [147], cost-effective approach for improving patient attendance [165], and more cost-effective compared with call reminders [174].

Not all trials have proven effective, however. Mobile technology did not improve asthma control or increase self-efficacy compared with paper-based monitoring, and there was no positive effect of SMS maintenance treatment on weight, eating behavior, or psychological well-being in obese children [158]. There was no significant reduction in nonattendance rates, as a result of texting appointment reminders to patients who persistently fail to attend their general practice appointments [148]. Also, the mobile technology was not cost-effective in one study [143]. As most interventions of these kinds in fact were effective it seems the reasons for not succeeding in some, relatively few, cases may have to do with local conditions.

The GIS technology in the context of health promotion and disease prevention was used for several purposes, including to measure distance between individuals’ area of residence and the location where each person used a computerized breast cancer education kiosk [131]; identify regional spots as potential territorial stations for a telemedicine service [181]; assess the neighborhood social and ecological contexts in mental health [182]; create a site selection strategy for the dissemination and pilot evaluation of a community-based fall prevention program for older adults [183]; and to analyze dental trauma using a GIS as a tool for integrating social, environmental, and epidemiological data [184]. In addition, GIS was used to discover the geographical variation of syphilis seeking clusters and hotspots [185]; plot measles cases on a digital map in real time [186]; visualize cancer risk patterns associated with incidence, mortality, and accessibility to care [187]; investigate factors associated with nosocomial transmission of resistant organisms [188]; and locate all out-of-hospital cardiac arrests (OHCAs) and identify clusters of OHCAs, as well as clusters of patients who did not receive bystander cardiopulmonary resuscitation [189]. In some studies, GIS was used to examine built environment characteristics and resident health behaviors as they relate to change in blood pressure [190]; quantify the effect of fish pond density on malaria occurrence [191]; identify geographic areas with elevated risk for the later development of amyotrophic lateral sclerosis among military personnel who served in the first Gulf War [192]; map the prevalence of malaria [193]; identify malaria hot spots [194]; create maps and charts displaying the geographic distribution of locations of injuries and their relationships with environmental and demographic parameters [195]; and examine sex-specific spatial patterns of overweight/obesity [196]. Some investigators, used GIS to explore the impact of the intervention coverage and the adherence to the intervention on malaria health outcome [197]; quantify the relationship between gonorrheal infection rates in California and a measure of poverty status and investigated how this relationship and the spatial dispersion of cases varied among the 4 dominant racial/ethnic groups [198]; develop spatial distribution maps of lymphatic filariasis in endemic areas [199]; and identify risk factors and affected areas of hantavirus infections in rodent hosts [200]. Furthermore, GIS was used to display the distribution of housing locations in relation to spatial dispersion, distress, stability, safety, and race/ethnic diversity for persons with psychiatric disabilities [201]; and assess the spatial distribution and associations for HIV testing and family planning use [202].

The use of GIS has enabled understanding of the interplay of psychological, social, environmental, area-level and sociodemographic influences on physical activity [203]; determination of actual travel time, and facilitation of the selection of community-based prevention program sites [183]. It has also enabled proving that space has an effect on outcome variables; mapping the prevalence of psychological distress, mental disorders, and use of mental health services and their correlates [182]; proving that there is significant variation in the occurrence of dental trauma [184]; enabled digital plotting cases of measles as they occurred in real time during the outbreak [186]; and finding significant relationships between the mapping of behavioral risk factors, health care services, transportation access, and policy advantages [187]. The technology was also used to demonstrated, by means of animated GIS, inappropriate patient placement for 19% of patients with methicillin-resistant Staphyloccocus aureus and insufficient time for hand hygiene in 14% (875/6248) of health care provider-patient contacts [188]; geographically map diabetes-risk scores and diabetes-screening rates [204]; and plot clusters of out-of-hospital cardiac arrests [189]. Through the mapping, fish pond density was found to be a significant predictor of malaria occurrence [191], and it was possible to see that service in particular locations of the Gulf was associated with an elevated risk for later developing amyotrophic lateral sclerosis, both before and after adjustment for branch of service and potential of exposure to chemical warfare agents in and around Khamisiyah, Iraq [192]. In addition, GIS identified 10 hot spots with extremely high risk of malaria and 14 hot spots with high risk of malaria [194]; enabled to create digital maps of injury spatial distribution and correlated injury type and location with patients’ clinical data [195]; enabled revealing marked geographical variation in overweight/obesity prevalence with higher values in the Northern and Atlantic health-regions and lower values in the southern and western health regions of Canada. Significant positive spatial autocorrelation was found for both males and females, with significant clusters of high values or hot spots of obesity in the Atlantic and Northern health regions of Alberta, Saskatchewan, Manitoba, and Ontario [196]; as indicated on the GIS maps, villages with malaria cases, lower intervention coverage, and lower adherence were identified. Although no malaria cases were detected in most villages with the best access to the district center, several cases were detected in the distal villages, where the intervention coverage and adherence to the intervention remained relatively lower [197]. The degree of spatial aggregation varied substantially among groups and was especially pronounced for African Americans with gonorrhea in the highest poverty category [198]; filaria monitoring visualization system maps demonstrated that filariasis remained unevenly distributed within districts [199]; land cover and elevation were found to be closely associated with the presence of hantavirus-infected rodent hosts [200]; maps displayed the distribution of housing locations in relation to spatial dispersion, distress, stability, safety, and racial/ethnic diversity and indicated that the developmental disabilities population in supportive housing was more spatially dispersed, and lived in less distressed, less unstable, more secure, but equally racially/ethnically diverse neighborhoods when compared with the psychiatric disabilities population in supportive housing [201]. Clustering among those using services is found as are spatial associations, indicating significant spatial variability in the utilization of health services [202].

#### Communication to or Between Health Care Providers

In this category the survey found 71 studies using mobile communication technologies with samples ranging from 8 to 4203, and 12 studies using GIS technologies. The interventions are described in Multimedia Appendices 8 and 9. Interventions using mobile communication technologies in both developed countries (Italy [205], US [1,92,206-228], UK [229-232], France [233], Canada [234-237], Japan [238], Australia [239], New Zealand [240], South Korea [241-245], and Denmark [246]), and developing ones (Thailand [247], Peru [248], Kenya [57,249,250], Taiwan [251,252], Israel [253-255], Uganda [256,257], Thailand [258], Singapore [259], Turkey [260], India [261], and South Africa [262]) have investigated the use of mobile phone-based apps, mobile phone voice call, SMS, and PDAs.

Interventions using mobile phone functions concerned providing automatic notification messages to the referring doctor and the consulted ophthalmologist on retinal diseases [205]; sending digital x-ray images via MMS [231,233,247]; and collecting patient diary information using an electronic peak flow meter linked to a mobile phone with an interactive screen to record current asthma symptoms transmitted to, and stored in, a server [229]. The video function was used to send reminders to assist with daily activities of persons with early dementia [230], and transmit teleconsultations, including clinical images of the amputated portion and stump as well as patient information between the physicians in the emergency room and the consultant plastic surgeon through Panasonic camera phones [251]. In addition, transmitting the 12-lead electrocardiography in an ambulance to the cell phone of the attendant emergency medical technician and then to the hospital and to cell phones of off-site cardiologists [252]. SMS was used to deliver diet, exercise, and behavior modification once a week to obese patients [241]. PDAs were used to track patient information during street outreach to the homeless in a major metropolitan area [208], and transmit radiological images to a remote physician [242].

Benefits of using mobile technologies include the possibility of identifying poor control more quickly and facilitating communication with health care professionals without the need for face-to-face consultation [229]. PDAs enabled clinicians to focus on building relationships instead of recreating documentation during patient encounters [208], and improve nursing work force, including accurate differential diagnosis and diagnostic reasoning, reduction of medication errors, reduction of health care costs, and development of effective treatment protocols [212]. A PDA-based technology providing behavioral messaging was an innovative, interesting, easy to use, educational, trustworthy, private, and nonjudgmental tool [248]. Teleconsultation using MMS is especially useful to improve remote management of orthopedic patients in local hospitals or for decisions of transfer when surgical treatment is needed [233]. SMS support had significantly improved ART adherence and rates of viral suppression compared with the control individuals [249], and enhanced chronic disease management support and patient-provider communications beyond the clinic setting [214].

Interventions using GIS technology in developed countries (US [263-267] and UK [268,269]) have used the technologies to map cancer rates and communicate the findings effectively [263]. This technology has also identified risk and developed potential interventions to address perinatal health problems [264], and developed a perinatal GIS model that helped community members to decide where to focus interventions and in continued use of GIS for planning [264].

#### Medical Education

On this topic the survey found eight studies using mobile communication technologies with samples ranging from 30 to 366. The interventions are described in Multimedia Appendix 10. All interventions used PDAs in developed countries (Austria [270], Canada [271], and US [272-274]). In these interventions the technology was used to enhance students' pharmacological and clinical contextual knowledge in both clinical practice and nursing education [270], and to determine a patient’s stage of change, providing scripted motivational interviews targeted to their stage, and making relevant health behavior and stage-based interventions immediately accessible [273]. In addition, a PDA was primarily used for personal apps by students during their preclinical training and as drug references and clinical calculators during their clinical training [209].

The use of PDA led to significant increase in self-efficacy [271]; PDAs were easy to use and perceived students’ use as beneficial to their learning in the clinical area [270]. A PDA-based tool did not increase key smoking cessation counseling behaviors compared with a paper-based reminder [273].

## Discussion

### Principal Findings

This research reviewed 271 articles on use of mobile technologies and GIS in improving health care. The articles were categorized into six predominant themes: treatment and disease management, data collection and disease surveillance, health support systems, health promotion and disease prevention, communication to or between health care providers, and medical education. Apps of GIS technology could be generally categorized into four predominant themes: disease surveillance, health support systems, health promotion and disease prevention, and communication to or between health care providers. These themes are not entirely distinct from one another and often overlap. For example, the use of GIS to examine the transmission of malaria is a disease surveillance app in that the spread of disease is mapped, but it is also a health support system in that disease spread is identified and tracked for the purpose of intervention development.

The overwhelming majority of the papers report positive results. Of course this is encouraging, but it also indicates some gaps in the body of research. First, it may mean that unsuccessful cases go unreported. Second, the papers we found focused on effects and tended not to discuss implementation issues. This means problems in making systems like the ones described implemented in the regular operations go unnoticed. This is an important gap as it may be an indication of a serious problem. Most studies in our sample are small-scale. It would of course be very useful to be able to scale up the small successful cases to a scale where more people could be helped and more health care could be afforded (as economic gains are among the positive results reported). Why has this not happened yet, despite all the positive results reported? From the papers reviewed here it is impossible to tell, but usually, as evidenced by a vast number of reports in the information systems literature, there are organizational issues behind such nondevelopment. Large-scale change is complicated, it requires aligning many actors, changing work procedures, standardizing of data, often some upfront investment in digitalization of data, legal, economic, and practical issues regarding communication and more. While these issues are much researched in other contexts, they are not yet researched in the particular contexts discussed in the papers reviewed here, there is a need to investigate implementation issues.

The findings also show that there is little integration between GIS and mobile technologies. While mobile technologies are successfully used for many types of interaction between patients and health care providers, there is little systematic use of operational data for strategic decision making. The communication yields large amounts of data, which can be analyzed so as to better understand how to design the communication effectively. This is not yet done, which indicates that the mobile systems are still pilots, not integrated in the organizations’ information infrastructure and not used for systematic monitoring, evaluation, and improvement of processes. This is a further urgent area for research and development. In order for health care processes to be effective they must integrate different kinds of existing technologies and data.

### Introduction of Apps

In all, the set of papers reviewed here reveal a trade in its very early beginning and in need of more systematic development. The review shows a large number of small-scale tests with little or no attempts to integrate the new technologies into standard operations. There are today a large number of apps, for mobile phones in the eHealth area, which are designed to support various needs related to supporting individuals in their handling of various health problems. Support includes tools for the patient’s own use, for collecting patient data for use by the doctor to support her analysis, and for communication between doctor and patient. Such apps are making their way into regular health care in many countries. This means data from mobile apps will increase in volume, which requires health care providers to find ways of systematically make use of incoming and outgoing data. Receiving and sending data effectively and reliably, quality control of data, security and privacy control, standardizing data formats for interoperability across systems, are examples of issues involved.

### Conclusions

In the cases reported here, the main technology is SMS or voice, mainly for the reason that these technologies are most widely available. But the need to develop effective communication and make use of data for process improvement remains the same whether the data comes from an SMS or a smartphone app. Also, the availability of smartphones are increasing everywhere, also in developing countries. This means there is a general need for research and development concerning integrating data from mobile apps into the back-office systems that make up the backbone of data handling in health care, and with systems that can analyze communication and provide support for improving processes.

## Multimedia Appendix 1

Treatment and disease management – overview of mHealth articles.

## Multimedia Appendix 2

Data collection and disease surveillance – overview of mHealth articles.

## Multimedia Appendix 3

Disease surveillance – overview of GIS articles.

## Multimedia Appendix 4

Health support systems – overview of mHealth articles.

## Multimedia Appendix 5

Health support systems – overview of GIS articles.

## Multimedia Appendix 6

Health promotion and disease prevention – overview of mHealth articles.

## Multimedia Appendix 7

Health promotion and disease prevention – overview of GIS articles.

## Multimedia Appendix 8

Communication to or between health care providers – overview of mHealth articles.

## Multimedia Appendix 9

Communication to or between health care providers – overview of GIS articles.

## Multimedia Appendix 10

Medical education – overview of articles mHealth articles.

## Abbreviations

ART: antiretroviral therapy
GIS: geographic information systems
IRS: insecticide treated nets and indoor residual spraying
MMS: multimedia messaging service
OHCA: out-of-hospital cardiac arrest
OLAP: online analytical processing
PDA: personal digital assistant
PP: paper-pencil
SMS: short message service

## References

[1] Puccio JA, Belzer M, Olson J, Martinez M, Salata C, Tucker D, Tanaka D (2006). The use of cell phone reminder calls for assisting HIV-infected adolescents and young adults to adhere to highly active antiretroviral therapy: a pilot study. AIDS Patient Care STDS.

[2] Geraghty M, Glynn F, Amin M, Kinsella J (2008). Patient mobile telephone 'text' reminder: a novel way to reduce non-attendance at the ENT out-patient clinic. J Laryngol Otol.

[3] Lester RT, Mills EJ, Kariri A, Ritvo P, Chung M, Jack W, Habyarimana J, Karanja S, Barasa S, Nguti R, Estambale B, Ngugi E, Ball TB, Thabane L, Kimani J, Gelmon L, Ackers M, Plummer FA (2009). The HAART cell phone adherence trial (WelTel Kenya1): a randomized controlled trial protocol. Trials.

[4] Mbuagbaw L, Thabane L, Ongolo-Zogo P, Lester RT, Mills E, Volmink J, Yondo D, Essi MJ, Bonono-Momnougui RC, Mba R, Ndongo JS, Nkoa FC, Ondoa HA (2011). The Cameroon Mobile Phone SMS (CAMPS) trial: a protocol for a randomized controlled trial of mobile phone text messaging versus usual care for improving adherence to highly active anti-retroviral therapy. Trials.

[5] Milne RG, Horne M, Torsney B (2006). SMS reminders in the UK national health service: an evaluation of its impact on "no-shows" at hospital out-patient clinics. Health Care Manage Rev.

[6] Strandbygaard U, Thomsen SF, Backer V (2010). A daily SMS reminder increases adherence to asthma treatment: a three-month follow-up study. Respir Med.

[7] Curioso WH, Alex Quistberg D, Cabello R, Gozzer E, Garcia PJ, Holmes KK, Kurth AE (2009). "It's time for your life": How should we remind patients to take medicines using short text messages?. AMIA Annu Symp Proc.

[8] Curioso WH, Kurth AE (2007). Access, use and perceptions regarding Internet, cell phones and PDAs as a means for health promotion for people living with HIV in Peru. BMC Med Inform Decis Mak.

[9] Montes JM, Medina E, Gomez-Beneyto M, Maurino J (2012). A short message service (SMS)-based strategy for enhancing adherence to antipsychotic medication in schizophrenia. Psychiatry Res.

[10] Ollivier L, Romand O, Marimoutou C, Michel R, Pognant C, Todesco A, Migliani R, Baudon D, Boutin JP (2009). Use of short message service (SMS) to improve malaria chemoprophylaxis compliance after returning from a malaria endemic area. Malar J.

[11] Sahm L, MacCurtain A, Hayden J, Roche C, Richards HL (2009). Electronic reminders to improve medication adherence--are they acceptable to the patient?. Pharm World Sci.

[12] Vervloet M, van Dijk L, Santen-Reestman J, van Vlijmen B, van Wingerden P, Bouvy ML, de Bakker DH (2012). SMS reminders improve adherence to oral medication in type 2 diabetes patients who are real time electronically monitored. Int J Med Inform.

[13] Vervloet M, van Dijk L, Santen-Reestman J, van Vlijmen B, Bouvy ML, de Bakker DH (2011). Improving medication adherence in diabetes type 2 patients through real time medication monitoring: a randomised controlled trial to evaluate the effect of monitoring patients' medication use combined with short message service (SMS) reminders. BMC Health Serv Res.

[14] Cho JH, Lee HC, Lim DJ, Kwon HS, Yoon KH (2009). Mobile communication using a mobile phone with a glucometer for glucose control in Type 2 patients with diabetes: as effective as an Internet-based glucose monitoring system. J Telemed Telecare.

[15] Curioso WH, Gozzer E, Valderrama M, Rodriguez-abad J, Villena JE, Villena AE, Peruana U, Heredia C (2009). Understanding the potential role of cell phones and the Internet to support care for diabetic patients and caregivers in Peru. American Medical Informatics Association Annual Symposium 2009: Biomedical and Health Informatics: From Foundations to Applications to Policy.

[16] Dick JJ, Nundy S, Solomon MC, Bishop KN, Chin MH, Peek ME (2011). Feasibility and usability of a text message-based program for diabetes self-management in an urban African-American population. J Diabetes Sci Technol.

[17] Faridi Z, Liberti L, Shuval K, Northrup V, Ali A, Katz DL (2008). Evaluating the impact of mobile telephone technology on type 2 diabetic patients' self-management: the NICHE pilot study. J Eval Clin Pract.

[18] Kim HS, Kim NC, Ahn SH (2006). Impact of a nurse short message service intervention for patients with diabetes. J Nurs Care Qual.

[19] Kollmann A, Riedl M, Kastner P, Schreier G, Ludvik B (2007). Feasibility of a mobile phone-based data service for functional insulin treatment of type 1 diabetes mellitus patients. J Med Internet Res.

[20] Logan AG, McIsaac WJ, Tisler A, Irvine MJ, Saunders A, Dunai A, Rizo CA, Feig DS, Hamill M, Trudel M, Cafazzo JA (2007). Mobile phone-based remote patient monitoring system for management of hypertension in diabetic patients. Am J Hypertens.

[21] Morak J, Schindler K, Goerzer E, Kastner P, Toplak H, Ludvik B, Schreier G (2008). A pilot study of mobile phone-based therapy for obese patients. J Telemed Telecare.

[22] Quinn CC, Gruber-Baldini AL, Shardell M, Weed K, Clough SS, Peeples M, Terrin M, Bronich-Hall L, Barr E, Lender D (2009). Mobile diabetes intervention study: testing a personalized treatment/behavioral communication intervention for blood glucose control. Contemp Clin Trials.

[23] Quinn CC, Shardell MD, Terrin ML, Barr EA, Ballew SH, Gruber-Baldini AL (2011). Cluster-randomized trial of a mobile phone personalized behavioral intervention for blood glucose control. Diabetes Care.

[24] Holtz B, Whitten P (2009). Managing asthma with mobile phones: a feasibility study. Telemed J E Health.

[25] Pinnock H, Slack R, Pagliari C, Price D, Sheikh A (2007). Understanding the potential role of mobile phone-based monitoring on asthma self-management: qualitative study. Clin Exp Allergy.

[26] Ryan D, Cobern W, Wheeler J, Price D, Tarassenko L (2005). Mobile phone technology in the management of asthma. J Telemed Telecare.

[27] Quinn CC, Clough SS, Minor JM, Lender D, Okafor MC, Gruber-Baldini A (2008). WellDoc mobile diabetes management randomized controlled trial: change in clinical and behavioral outcomes and patient and physician satisfaction. Diabetes Technol Ther.

[28] Boker A, Feetham HJ, Armstrong A, Purcell P, Jacobe H (2012). Do automated text messages increase adherence to acne therapy? Results of a randomized, controlled trial. J Am Acad Dermatol.

[29] Dowshen N, Kuhns LM, Johnson A, Holoyda BJ, Garofalo R (2012). Improving adherence to antiretroviral therapy for youth living with HIV/AIDS: a pilot study using personalized, interactive, daily text message reminders. J Med Internet Res.

[30] MacDonell K, Gibson-Scipio W, Lam P, Naar-King S, Chen X (2012). Text messaging to measure asthma medication use and symptoms in urban African American emerging adults: a feasibility study. J Asthma.

[31] Foreman KF, Stockl KM, Le LB, Fisk E, Shah SM, Lew HC, Solow BK, Curtis BS (2012). Impact of a text messaging pilot program on patient medication adherence. Clin Ther.

[32] Lee HJ, Lee SH, Ha KS, Jang HC, Chung WY, Kim JY, Chang YS, Yoo DH (2009). Ubiquitous healthcare service using Zigbee and mobile phone for elderly patients. Int J Med Inform.

[33] Cocosila M, Archer N, Haynes RB, Yuan Y (2009). Can wireless text messaging improve adherence to preventive activities? Results of a randomised controlled trial. Int J Med Inform.

[34] Kelly JD, Giordano TP (2011). Mobile phone technologies improve adherence to antiretroviral treatment in a resource-limited setting: a randomized controlled trial of text message reminders. AIDS.

[35] Mbuagbaw L, Thabane L, Ongolo-Zogo P, Lester RT, Mills EJ, Smieja M, Dolovich L, Kouanfack C (2012). The Cameroon Mobile Phone SMS (CAMPS) trial: a randomized trial of text messaging versus usual care for adherence to antiretroviral therapy. PLoS One.

[36] da Costa TM, Barbosa BJ, Gomes e Costa DA, Sigulem D, de Fátima Marin H, Filho AC, Pisa IT (2012). Results of a randomized controlled trial to assess the effects of a mobile SMS-based intervention on treatment adherence in HIV/AIDS-infected Brazilian women and impressions and satisfaction with respect to incoming messages. Int J Med Inform.

[37] Petrie KJ, Perry K, Broadbent E, Weinman J (2012). A text message programme designed to modify patients' illness and treatment beliefs improves self-reported adherence to asthma preventer medication. Br J Health Psychol.

[38] Geraghty M, Glynn F, Amin M, Kinsella J (2008). Patient mobile telephone 'text' reminder: a novel way to reduce non-attendance at the ENT out-patient clinic. J Laryngol Otol.

[39] Bexelius C, Merk H, Sandin S, Ekman A, Nyrén O, Kühlmann-Berenzon S, Linde A, Litton JE (2009). SMS versus telephone interviews for epidemiological data collection: feasibility study estimating influenza vaccination coverage in the Swedish population. Eur J Epidemiol.

[40] Dale O, Hagen KB (2007). Despite technical problems personal digital assistants outperform pen and paper when collecting patient diary data. J Clin Epidemiol.

[41] Heiberg T, Kvien TK, Dale Ø, Mowinckel P, Aanerud GJ, Songe-Møller AB, Uhlig T, Hagen KB (2007). Daily health status registration (patient diary) in patients with rheumatoid arthritis: a comparison between personal digital assistant and paper-pencil format. Arthritis Rheum.

[42] Fowles ER, Gentry B (2008). The feasibility of personal digital assistants (PDAs) to collect dietary intake data in low-income pregnant women. J Nutr Educ Behav.

[43] Galliher JM, Stewart TV, Pathak PK, Werner JJ, Dickinson LM, Hickner JM (2008). Data collection outcomes comparing paper forms with PDA forms in an office-based patient survey. Ann Fam Med.

[44] Siracuse MV, Sowell JG (2008). Doctor of pharmacy students' use of personal digital assistants. Am J Pharm Educ.

[45] Magee M, Isakov A, Paradise HT, Sullivan P (2011). Mobile phones and short message service texts to collect situational awareness data during simulated public health critical events. Am J Disaster Med.

[46] Homayounfar K, Spiller J, von Stillfried F, Raible M (2007). [Mobile and digital documentation of inpatient treatments : use of personal digital assistants in addition with the ClinicCoach(c) software]. [article in German]. Unfallchirurg.

[47] MacGregor DL, Tallett S, MacMillan S, Gerber R, O'Brodovich H (2006). Clinical and education workload measurements using personal digital assistant-based software. Pediatrics.

[48] Raybardhan S, Balen RM, Partovi N, Loewen P, Liu G, Jewesson PJ (2005). Documenting drug-related problems with personal digital assistants in a multisite health system. Am J Health Syst Pharm.

[49] Whitford HM, Donnan PT, Symon AG, Kellett G, Monteith-Hodge E, Rauchhaus P, Wyatt JC (2012). Evaluating the reliability, validity, acceptability, and practicality of SMS text messaging as a tool to collect research data: results from the Feeding Your Baby project. J Am Med Inform Assoc.

[50] Tasker AP, Gibson L, Franklin V, Gregor P, Greene S (2007). What is the frequency of symptomatic mild hypoglycemia in type 1 diabetes in the young?: assessment by novel mobile phone technology and computer-based interviewing. Pediatr Diabetes.

[51] Scherr D, Zweiker R, Kollmann A, Kastner P, Schreier G, Fruhwald FM (2006). Mobile phone-based surveillance of cardiac patients at home. J Telemed Telecare.

[52] Wang DH, Kogashiwa M, Kira S (2006). Development of a new instrument for evaluating individuals' dietary intakes. J Am Diet Assoc.

[53] Kikunaga S, Tin T, Ishibashi G, Wang DH, Kira S (2007). The application of a handheld personal digital assistant with camera and mobile phone card (Wellnavi) to the general population in a dietary survey. J Nutr Sci Vitaminol (Tokyo).

[54] Andreatta P, Debpuur D, Danquah A, Perosky J (2011). Using cell phones to collect postpartum hemorrhage outcome data in rural Ghana. Int J Gynaecol Obstet.

[55] Auld AF, Wambua N, Onyango J, Marston B, Namulanda G, Ackers M, Oluoch T, Karisa A, Hightower A, Shiraishi RW, Nakashima A, Sitienei J (2010). Piloting the use of personal digital assistants for tuberculosis and human immunodeficiency virus surveillance, Kenya, 2007. Int J Tuberc Lung Dis.

[56] Pierre S, Emukule G, Wambugu S, Kabore I, Mwarogo P (2009). A comparison of paper-based questionnaires with PDA for behavioral surveys in Africa : Findings from a behavioral monitoring survey in Kenya. J Health Inform Dev Ctries.

[57] Diero L, Rotich JK, Bii J, Mamlin BW, Einterz RM, Kalamai IZ, Tierney WM (2006). A computer-based medical record system and personal digital assistants to assess and follow patients with respiratory tract infections visiting a rural Kenyan health centre. BMC Med Inform Decis Mak.

[58] Bernabe-Ortiz A, Curioso WH, Gonzales MA, Evangelista W, Castagnetto JM, Carcamo CP, Hughes JP, Garcia PJ, Garnett GP, Holmes KK (2008). Handheld computers for self-administered sensitive data collection: a comparative study in Peru. BMC Med Inform Decis Mak.

[59] Blaya JA, Gomez W, Rodriguez P, Fraser H (2008). Cost and implementation analysis of a personal digital assistant system for laboratory data collection. Int J Tuberc Lung Dis.

[60] Blaya JA, Cohen T, Rodríguez P, Kim J, Fraser HS (2009). Personal digital assistants to collect tuberculosis bacteriology data in Peru reduce delays, errors, and workload, and are acceptable to users: cluster randomized controlled trial. Int J Infect Dis.

[61] Cheng KG, Ernesto F, Ovalle-Bahamón RE, Truong KN (2011). Barriers to acceptance of personal digital assistants for HIV/AIDS data collection in Angola. Int J Med Inform.

[62] Jian WS, Hsu MH, Sukati H, Syed-Abdul S, Scholl J, Dube N, Hsu CK, Wu TJ, Lin V, Chi T, Chang P, Li YC (2012). LabPush: a pilot study of providing remote clinics with laboratory results via short message service (SMS) in Swaziland, Africa. PLoS One.

[63] Kew S (2010). Text messaging: an innovative method of data collection in medical research. BMC Res Notes.

[64] Patnaik S, Brunskill E, Thies W (2009). Evaluating the accuracy of data collection on mobile phones: A study of forms, SMS. ICTD'09 Proceedings of the 3rd international conference on Information and communication technologies and development.

[65] Ganesan M, Prashant S, Pushpa V, Janakiraman N (2011). The use of mobile phones as a tool for capturing patient data in Southern Rural Tamil Nadu , India. J Health Inform Dev Ctries.

[66] Safaie A, Mousavi SM, LaPorte RE, Goya MM, Zahraie M (2006). Introducing a model for communicable diseases surveillance: cell phone surveillance (CPS). Eur J Epidemiol.

[67] Yang C, Yang J, Luo X, Gong P (2009). Use of mobile phones in an emergency reporting system for infectious disease surveillance after the Sichuan earthquake in China. Bull World Health Organ.

[68] Rudkin SE, Langdorf MI, Macias D, Oman JA, Kazzi AA (2006). Personal digital assistants change management more often than paper texts and foster patient confidence. Eur J Emerg Med.

[69] Gosselin P, Lebel G, Rivest S, Douville-Fradet M (2005). The Integrated System for Public Health Monitoring of West Nile Virus (ISPHM-WNV): a real-time GIS for surveillance and decision-making. Int J Health Geogr.

[70] Flahault A, Blanchon T, Dorléans Y, Toubiana L, Vibert JF, Valleron AJ (2006). Virtual surveillance of communicable diseases: a 20-year experience in France. Stat Methods Med Res.

[71] Horst MA, Coco AS (2010). Observing the spread of common illnesses through a community: using Geographic Information Systems (GIS) for surveillance. J Am Board Fam Med.

[72] Kim AA, Martinez AN, Klausner JD, Goldenson J, Kent C, Liska S, McFarland W (2009). Use of sentinel surveillance and geographic information systems to monitor trends in HIV prevalence, incidence, and related risk behavior among women undergoing syphilis screening in a jail setting. J Urban Health.

[73] Vanmeulebrouk B, Rivett U, Ricketts A, Loudon M (2008). Open source GIS for HIV/AIDS management. Int J Health Geogr.

[74] Cinnamon J, Schuurman N (2010). Injury surveillance in low-resource settings using Geospatial and Social Web technologies. Int J Health Geogr.

[75] Chang AY, Parrales ME, Jimenez J, Sobieszczyk ME, Hammer SM, Copenhaver DJ, Kulkarni RP (2009). Combining Google Earth and GIS mapping technologies in a dengue surveillance system for developing countries. Int J Health Geogr.

[76] Kandwal R, Garg PK, Garg RD (2009). Health GIS and HIV/AIDS studies: perspective and retrospective. J Biomed Inform.

[77] Kelly GC, Hii J, Batarii W, Donald W, Hale E, Nausien J, Pontifex S, Vallely A, Tanner M, Clements A (2010). Modern geographical reconnaissance of target populations in malaria elimination zones. Malar J.

[78] Messina JP, Emch M, Muwonga J, Mwandagalirwa K, Edidi SB, Mama N, Okenge A, Meshnick SR (2010). Spatial and socio-behavioral patterns of HIV prevalence in the Democratic Republic of Congo. Soc Sci Med.

[79] Mungrue K, Mahabir R (2011). The rabies epidemic in Trinidad of 1923 to 1937: an evaluation with a Geographic Information System. Wilderness Environ Med.

[80] Kelly GC, Hale E, Donald W, Batarii W, Bugoro H, Nausien J, Smale J, Palmer K, Bobogare A, Taleo G, Vallely A, Tanner M, Vestergaard LS, Clements AC (2013). A high-resolution geospatial surveillance-response system for malaria elimination in Solomon Islands and Vanuatu. Malar J.

[81] Arsand E, Tufano JT, Ralston JD, Hjortdahl P (2008). Designing mobile dietary management support technologies for people with diabetes. J Telemed Telecare.

[82] Brendryen H, Drozd F, Kraft P (2008). A digital smoking cessation program delivered through internet and cell phone without nicotine replacement (happy ending): randomized controlled trial. J Med Internet Res.

[83] Benhamou PY, Melki V, Boizel R, Perreal F, Quesada JL, Bessieres-Lacombe S, Bosson JL, Halimi S, Hanaire H (2007). One-year efficacy and safety of Web-based follow-up using cellular phone in type 1 diabetic patients under insulin pump therapy: the PumpNet study. Diabetes Metab.

[84] Brock TP, Smith SR (2007). Using digital videos displayed on personal digital assistants (PDAs) to enhance patient education in clinical settings. Int J Med Inform.

[85] Franklin VL, Waller A, Pagliari C, Greene SA (2006). A randomized controlled trial of Sweet Talk, a text-messaging system to support young people with diabetes. Diabet Med.

[86] Free C, Whittaker R, Knight R, Abramsky T, Rodgers A, Roberts IG (2009). Txt2stop: a pilot randomised controlled trial of mobile phone-based smoking cessation support. Tob Control.

[87] Free C, Knight R, Robertson S, Whittaker R, Edwards P, Zhou W, Rodgers A, Cairns J, Kenward MG, Roberts I (2011). Smoking cessation support delivered via mobile phone text messaging (txt2stop): a single-blind, randomised trial. Lancet.

[88] Naughton F, Prevost AT, Gilbert H, Sutton S (2012). Randomized controlled trial evaluation of a tailored leaflet and SMS text message self-help intervention for pregnant smokers (MiQuit). Nicotine Tob Res.

[89] Waller A, Franklin V, Pagliari C, Greene S (2006). Participatory design of a text message scheduling system to support young people with diabetes. Health Informatics J.

[90] De Toledo P, Lalinde W, Del Pozo F, Thurber D, Jimenez-Fernandez S (2006). Interoperability of a mobile health care solution with electronic healthcare record systems. Conf Proc IEEE Eng Med Biol Soc.

[91] Epstein RH, Ekbatani A, Kaplan J, Shechter R, Grunwald Z (2010). Development of a staff recall system for mass casualty incidents using cell phone text messaging. Anesth Analg.

[92] Hanauer DA, Wentzell K, Laffel N, Laffel LM (2009). Computerized Automated Reminder Diabetes System (CARDS): e-mail and SMS cell phone text messaging reminders to support diabetes management. Diabetes Technol Ther.

[93] Patrick K, Raab F, Adams MA, Dillon L, Zabinski M, Rock CL, Griswold WG, Norman GJ (2009). A text message-based intervention for weight loss: randomized controlled trial. J Med Internet Res.

[94] Riley W, Obermayer J, Jean-Mary J (2008). Internet and mobile phone text messaging intervention for college smokers. J Am Coll Health.

[95] Sevick MA, Zickmund S, Korytkowski M, Piraino B, Sereika S, Mihalko S, Snetselaar L, Stumbo P, Hausmann L, Ren D, Marsh R, Sakraida T, Gibson J, Safaien M, Starrett TJ, Burke LE (2008). Design, feasibility, and acceptability of an intervention using personal digital assistant-based self-monitoring in managing type 2 diabetes. Contemp Clin Trials.

[96] Ybarra ML, Holtrop JS, Bağci Bosi AT, Emri S (2012). Design considerations in developing a text messaging program aimed at smoking cessation. J Med Internet Res.

[97] Uhrig JD, Lewis MA, Bann CM, Harris JL, Furberg RD, Coomes CM, Kuhns LM (2012). Addressing HIV knowledge, risk reduction, social support, and patient involvement using SMS: results of a proof-of-concept study. J Health Commun.

[98] Johansson PE, Petersson GI, Nilsson GC (2010). Personal digital assistant with a barcode reader--a medical decision support system for nurses in home care. Int J Med Inform.

[99] Kouris I, Mougiakakou S, Scarnato L, Iliopoulou D, Diem P, Vazeou A, Koutsouris D (2010). Mobile phone technologies and advanced data analysis towards the enhancement of diabetes self-management. Int J Electron Healthc.

[100] te Boveldt N, Engels Y, Besse K, Vissers K, Vernooij-Dassen M (2011). Rationale, design, and implementation protocol of the Dutch clinical practice guideline pain in patients with cancer: a cluster randomised controlled trial with Short Message Service (SMS) and Interactive Voice Response (IVR). Implement Sci.

[101] Triventi M, Mattei E, Censi F, Calcagnini G, Mastrantonio F, Giansanti D, Maccioni G, Macellari V, BartoliniMS-based platform for cardiovascular tele-monitoring (2008). SMS-based platform for cardiovascular tele-monitoring. Computers in Cardiology, 2008.

[102] Whittaker R, Dorey E, Bramley D, Bullen C, Denny S, Elley CR, Maddison R, McRobbie H, Parag V, Rodgers A, Salmon P (2011). A theory-based video messaging mobile phone intervention for smoking cessation: randomized controlled trial. J Med Internet Res.

[103] Whittaker R, Maddison R, McRobbie H, Bullen C, Denny S, Dorey E, Ellis-Pegler M, van Rooyen J, Rodgers A (2008). A multimedia mobile phone-based youth smoking cessation intervention: findings from content development and piloting studies. J Med Internet Res.

[104] Whittaker R, Merry S, Stasiak K, McDowell H, Doherty I, Shepherd M, Dorey E, Parag V, Ameratunga S, Rodgers A (2012). MEMO--a mobile phone depression prevention intervention for adolescents: development process and postprogram findings on acceptability from a randomized controlled trial. J Med Internet Res.

[105] Choi J, Yoo S, Park H, Chun J (2006). MobileMed: a PDA-based mobile clinical information system. IEEE Trans Inf Technol Biomed.

[106] Altuwaijri MM, Sughayr AM, Hassan MA, Alazwari FM (2012). The effect of integrating short messaging services` reminders with electronic medical records on non-attendance rates. Saudi Med J.

[107] Barrington J, Wereko-Brobby O, Ward P, Mwafongo W, Kungulwe S (2010). SMS for Life: a pilot project to improve anti-malarial drug supply management in rural Tanzania using standard technology. Malar J.

[108] Chau S, Turner P (2006). Utilisation of mobile handheld devices for care management at an Australian aged care facility. Electron Commer Res Appl.

[109] Blaya J, Fraser HS (2006). Development, implementation and preliminary study of a PDA-based tuberculosis result collection system. AMIA Annu Symp Proc.

[110] Curioso WH, Karras BT, Campos PE, Buendia C, Holmes KK, Kimball AM (2005). Design and implementation of Cell-PREVEN: a real-time surveillance system for adverse events using cell phones in Peru. AMIA Annu Symp Proc.

[111] Kurth AE, Curioso WH, Ngugi E, McClelland L, Segura P, Cabello R, Berry DL (2007). Personal digital assistants for HIV treatment adherence, safer sex behavior support, and provider training in resource-constrained settings. AMIA Annu Symp Proc.

[112] Dwolatzky B, Trengove E, Struthers H, McIntyre JA, Martinson NA (2006). Linking the global positioning system (GPS) to a personal digital assistant (PDA) to support tuberculosis control in South Africa: a pilot study. Int J Health Geogr.

[113] Ho MR, Owusu EK, Aoki PM (2009). Claim Mobile: engaging conflicting stakeholder requirements in healthcare in Uganda. International Conference on Information and Communication Technologies and Development.

[114] Mao Y, Zhang Y, Zhai S (2008). Mobile phone text messaging for pharmaceutical care in a hospital in China. J Telemed Telecare.

[115] Yu P, de Courten M, Pan E, Galea G, Pryor J (2009). The development and evaluation of a PDA-based method for public health surveillance data collection in developing countries. Int J Med Inform.

[116] Mohan P, Marin D, Sultan S, Deen A (2008). MediNet: personalizing the self-care process for patients with diabetes and cardiovascular disease using mobile telephony. Conf Proc IEEE Eng Med Biol Soc.

[117] Onono MA, Carraher N, Cohen RC, Bukusi EA, Turan JM (2011). Use of personal digital assistants for data collection in a multi-site AIDS stigma study in rural south Nyanza, Kenya. Afr Health Sci.

[118] Shet A, Arumugam K, Rodrigues R, Rajagopalan N, Shubha K, Raj T, D'souza G, De Costa A (2010). Designing a mobile phone-based intervention to promote adherence to antiretroviral therapy in South India. AIDS Behav.

[119] Baum S, Kendall E, Muenchberger H, Gudes O, Yigitcanlar T (2010). Geographical information systems: an effective planning and decision-making platform for community health coalitions in Australia. HIM J.

[120] Gudes O, Pathak V, Kendall E, Yigitcanlar T, Traver V, Filipe J, Gamboa H (2011). Thinking spatially, acting collaboratively&amp;#8239;: a GIS-based health decision support system for improving the collaborative health-planning practice. HEALTHINF 2011 - Proceedings of the International Conference on Health Informatics.

[121] Boulos MN, Russell C, Smith M (2005). Web GIS in practice II: interactive SVG maps of diagnoses of sexually transmitted diseases by Primary Care Trust in London, 1997 - 2003. Int J Health Geogr.

[122] Choi M, Afzal B, Sattler B (2006). Geographic information systems: a new tool for environmental health assessments. Public Health Nurs.

[123] Scotch M, Parmanto B, Monaco V (2008). Evaluation of SOVAT: an OLAP-GIS decision support system for community health assessment data analysis. BMC Med Inform Decis Mak.

[124] Gao S, Mioc D, Anton F, Yi X, Coleman DJ (2008). Online GIS services for mapping and sharing disease information. Int J Health Geogr.

[125] Schuurman N, Randall E, Berube M (2011). A spatial decision support tool for estimating population catchments to aid rural and remote health service allocation planning. Health Informatics J.

[126] Jean-Baptiste R, Toubiana L, Le Mignot L, Ben Said M, Mugnier C, Le Bihan-Benjamin C, Jaïs JP, Landais P (2005). A Web-based GIS for health care decision-support. AMIA Annu Symp Proc.

[127] Chanda E, Mukonka VM, Mthembu D, Kamuliwo M, Coetzer S, Shinondo CJ (2012). Using a geographical-information-system-based decision support to enhance malaria vector control in zambia. J Trop Med.

[128] Kelly GC, Seng CM, Donald W, Taleo G, Nausien J, Batarii W, Iata H, Tanner M, Vestergaard LS, Clements AC (2011). A spatial decision support system for guiding focal indoor residual interventions in a malaria elimination zone. Geospat Health.

[129] Lozano-Fuentes S, Elizondo-Quiroga D, Farfan-Ale JA, Loroño-Pino MA, Garcia-Rejon J, Gomez-Carro S, Lira-Zumbardo V, Najera-Vazquez R, Fernandez-Salas I, Calderon-Martinez J, Dominguez-Galera M, Mis-Avila P, Morris N, Coleman M, Moore CG, Beaty BJ, Eisen L (2008). Use of Google Earth to strengthen public health capacity and facilitate management of vector-borne diseases in resource-poor environments. Bull World Health Organ.

[130] Murad AA (2007). Creating a GIS application for health services at Jeddah city. Comput Biol Med.

[131] Alcaraz KI, Kreuter MW, Bryan RP (2009). Use of GIS to identify optimal settings for cancer prevention and control in African American communities. Prev Med.

[132] Armstrong AW, Watson AJ, Makredes M, Frangos JE, Kimball AB, Kvedar JC (2009). Text-message reminders to improve sunscreen use: a randomized, controlled trial using electronic monitoring. Arch Dermatol.

[133] Stockwell MS, Kharbanda EO, Martinez RA, Lara M, Vawdrey D, Natarajan K, Rickert VI (2012). Text4Health: impact of text message reminder-recalls for pediatric and adolescent immunizations. Am J Public Health.

[134] Carroll AE, Marrero DG, Downs SM (2007). The HealthPia GlucoPack Diabetes phone: a usability study. Diabetes Technol Ther.

[135] Schembre SM, Yuen J (2011). Project TwEATs. A feasibility study testing the use of automated text messaging to monitor appetite ratings in a free-living population. Appetite.

[136] Woolford SJ, Clark SJ, Strecher VJ, Resnicow K (2010). Tailored mobile phone text messages as an adjunct to obesity treatment for adolescents. J Telemed Telecare.

[137] Vidrine DJ, Arduino RC, Lazev AB, Gritz ER (2006). A randomized trial of a proactive cellular telephone intervention for smokers living with HIV/AIDS. AIDS.

[138] Vidrine D, Arduino R, Gritz E (2006). Impact of a cell phone intervention on mediating mechanisms of smoking cessation in individuals living with HIV/AIDS. Nicotine Tob Res.

[139] Gerber BS, Stolley MR, Thompson AL, Sharp LK, Fitzgibbon ML (2009). Mobile phone text messaging to promote healthy behaviors and weight loss maintenance: a feasibility study. Health Informatics J.

[140] Stockwell MS, Kharbanda EO, Martinez RA, Vargas CY, Vawdrey DK, Camargo S (2012). Effect of a text messaging intervention on influenza vaccination in an urban, low-income pediatric and adolescent population: a randomized controlled trial. JAMA.

[141] Odeny TA, Bailey RC, Bukusi EA, Simoni JM, Tapia KA, Yuhas K, Holmes KK, McClelland RS (2012). Text messaging to improve attendance at post-operative clinic visits after adult male circumcision for HIV prevention: a randomized controlled trial. PLoS One.

[142] Menon-Johansson AS, McNaught F, Mandalia S, Sullivan AK (2006). Texting decreases the time to treatment for genital Chlamydia trachomatis infection. Sex Transm Infect.

[143] Ryan D, Price D, Musgrave SD, Malhotra S, Lee AJ, Ayansina D, Sheikh A, Tarassenko L, Pagliari C, Pinnock H (2012). Clinical and cost effectiveness of mobile phone supported self monitoring of asthma: multicentre randomised controlled trial. BMJ.

[144] Farmer A, Gibson O, Hayton P, Bryden K, Dudley C, Neil A, Tarassenko L (2005). A real-time, mobile phone-based telemedicine system to support young adults with type 1 diabetes. Inform Prim Care.

[145] Miskelly F (2005). Electronic tracking of patients with dementia and wandering using mobile phone technology. Age Ageing.

[146] Robinson S, Perkins S, Bauer S, Hammond N, Treasure J, Schmidt U (2006). Aftercare intervention through text messaging in the treatment of bulimia nervosa--feasibility pilot. Int J Eat Disord.

[147] Brannan SO, Dewar C, Taggerty L, Clark S (2011). The effect of short messaging service text on non-attendance in a general ophthalmology clinic. Scott Med J.

[148] Fairhurst K, Sheikh A (2008). Texting appointment reminders to repeated non-attenders in primary care: randomised controlled study. Qual Saf Health Care.

[149] Foley J, O'Neill M (2009). Use of mobile telephone short message service (SMS) as a reminder: the effect on patient attendance. Eur Arch Paediatr Dent.

[150] Koshy E, Car J, Majeed A (2008). Effectiveness of mobile-phone short message service (SMS) reminders for ophthalmology outpatient appointments: observational study. BMC Ophthalmol.

[151] Kruse LV, Hansen LG, Olesen C (2009). [Non-attendance at a pediatric outpatient clinic. SMS text messaging improves attendance]. [article in Danish]. Ugeskr Laeger.

[152] Kunutsor S, Walley J, Katabira E, Muchuro S, Balidawa H, Namagala E, Ikoona E (2010). Using mobile phones to improve clinic attendance amongst an antiretroviral treatment cohort in rural Uganda: a cross-sectional and prospective study. AIDS Behav.

[153] Perry JG (2011). A preliminary investigation into the effect of the use of the short message service (SMS) on patient attendance at an NHS Dental Access Centre in Scotland. Prim Dent Care.

[154] Sims H, Sanghara H, Hayes D, Wandiembe S, Finch M, Jakobsen H, Tsakanikos E, Okocha CI, Kravariti E (2012). Text message reminders of appointments: a pilot intervention at four community mental health clinics in London. Psychiatr Serv.

[155] Gammon D, Arsand E, Walseth OA, Andersson N, Jenssen M, Taylor T (2005). Parent-child interaction using a mobile and wireless system for blood glucose monitoring. J Med Internet Res.

[156] Dalton AF, Ní Scanaill C, Carew S, Lyons D, OLaighin G (2007). A clinical evaluation of a remote mobility monitoring system based on SMS messaging. Conf Proc IEEE Eng Med Biol Soc.

[157] Scanaill CN, Ahearne B, Lyons GM (2006). Long-term telemonitoring of mobility trends of elderly people using SMS messaging. IEEE Trans Inf Technol Biomed.

[158] de Niet J, Timman R, Bauer S, van den Akker E, Buijks H, de Klerk C, Kordy H, Passchier J (2012). The effect of a short message service maintenance treatment on body mass index and psychological well-being in overweight and obese children: a randomized controlled trial. Pediatr Obes.

[159] de Niet J, Timman R, Bauer S, van den Akker E, de Klerk C, Kordy H, Passchier J (2012). Short message service reduces dropout in childhood obesity treatment: a randomized controlled trial. Health Psychol.

[160] Hayn D, Koller S, Hofmann-Wellenhof R, Salmhofer W, Kastner P, Schreier G (2009). Mobile phone-based teledermatologic compliance management - preliminary results of the TELECOMP study. Stud Health Technol Inform.

[161] Bramley D, Riddell T, Whittaker R, Corbett T, Lin R-B, Wills M, Jones M, Rodgers A (2005). Smoking cessation using mobile phone text messaging is as effective in Maori as non-Maori. N Z Med J.

[162] Rodgers A, Corbett T, Bramley D, Riddell T, Wills M, Lin RB, Jones M (2005). Do u smoke after txt? Results of a randomised trial of smoking cessation using mobile phone text messaging. Tob Control.

[163] Gold J, Lim MS, Hocking JS, Keogh LA, Spelman T, Hellard ME (2011). Determining the impact of text messaging for sexual health promotion to young people. Sex Transm Dis.

[164] Downer SR, Meara JG, Da Costa AC (2005). Use of SMS text messaging to improve outpatient attendance. Med J Aust.

[165] Downer SR, Meara JG, Da Costa AC, Sethuraman K (2006). SMS text messaging improves outpatient attendance. Aust Health Rev.

[166] Kim HS (2007). A randomized controlled trial of a nurse short-message service by cellular phone for people with diabetes. Int J Nurs Stud.

[167] Kim H-S, Jeong H-S (2007). A nurse short message service by cellular phone in type-2 diabetic patients for six months. J Clin Nurs.

[168] Zurovac D, Sudoi RK, Akhwale WS, Ndiritu M, Hamer DH, Rowe AK, Snow RW (2011). The effect of mobile phone text-message reminders on Kenyan health workers' adherence to malaria treatment guidelines: a cluster randomised trial. Lancet.

[169] Ostojic V, Cvoriscec B, Ostojic SB, Reznikoff D, Stipic-Markovic A, Tudjman Z (2005). Improving asthma control through telemedicine: a study of short-message service. Telemed J E Health.

[170] Prabhakaran L, Chee WY, Chua KC, Abisheganaden J, Wong WM (2010). The use of text messaging to improve asthma control: a pilot study using the mobile phone short messaging service (SMS). J Telemed Telecare.

[171] da Costa TM, Salomão PL, Martha AS, Pisa IT, Sigulem D (2010). The impact of short message service text messages sent as appointment reminders to patients' cell phones at outpatient clinics in São Paulo, Brazil. Int J Med Inform.

[172] Lin H, Chen W, Luo L, Congdon N, Zhang X, Zhong X, Liu Z, Chen W, Wu C, Zheng D, Deng D, Ye S, Lin Z, Zou X, Liu Y (2012). Effectiveness of a short message reminder in increasing compliance with pediatric cataract treatment: a randomized trial. Ophthalmology.

[173] Engineers IOEAE (2008). Design of wireless mobile monitoring of blood pressure for underserved in China by using short messaging service. 2008 International Special Topic Conference on Information Technology and Applications in Biomedicine.

[174] Chen ZW, Fang LZ, Chen LY, Dai HL (2008). Comparison of an SMS text messaging and phone reminder to improve attendance at a health promotion center: a randomized controlled trial. J Zhejiang Univ Sci B.

[175] De Costa A, Shet A, Kumarasamy N, Ashorn P, Eriksson B, Bogg L, Diwan V, HIVIND study team (2010). Design of a randomized trial to evaluate the influence of mobile phone reminders on adherence to first line antiretroviral treatment in South India--the HIVIND study protocol. BMC Med Res Methodol.

[176] Khokhar A (2009). Short text messages (SMS) as a reminder system for making working women from Delhi Breast Aware. Asian Pac J Cancer Prev.

[177] Prasad S, Anand R (2012). Use of mobile telephone short message service as a reminder: the effect on patient attendance. Int Dent J.

[178] Leong KC, Chen WS, Leong KW, Mastura I, Mimi O, Sheikh MA, Zailinawati AH, Ng CJ, Phua KL, Teng CL (2006). The use of text messaging to improve attendance in primary care: a randomized controlled trial. Fam Pract.

[179] Liew SM, Tong SF, Lee VK, Ng CJ, Leong KC, Teng CL (2009). Text messaging reminders to reduce non-attendance in chronic disease follow-up: a clinical trial. Br J Gen Pract.

[180] Bielli E, Carminati F, La Capra S, Lina M, Brunelli C, Tamburini M (2004). A Wireless Health Outcomes Monitoring System (WHOMS): development and field testing with cancer patients using mobile phones. BMC Med Inform Decis Mak.

[181] Bramanti A, Bonanno L, Celona A, Bertuccio S, Calisto A, Lanzafame P, Bramanti P (2010). GIS and spatial analysis for costs and services optimization in neurological telemedicine. Conf Proc IEEE Eng Med Biol Soc.

[182] Caron J, Fleury MJ, Perreault M, Crocker A, Tremblay J, Tousignant M, Kestens Y, Cargo M, Daniel M (2012). Prevalence of psychological distress and mental disorders, and use of mental health services in the epidemiological catchment area of Montreal South-West. BMC Psychiatry.

[183] Carlson T, York S, Primomo J (2011). The utilization of geographic information systems to create a site selection strategy to disseminate an older adult fall prevention program. Soc Sci J.

[184] Carvalho ML, Moysés SJ, Bueno RE, Shimakura S, Moysés ST (2010). A geographical population analysis of dental trauma in school-children aged 12 and 15 in the city of Curitiba-Brazil. BMC Health Serv Res.

[185] Cheng YJ, Norris J, Bao CJ, Liang Q, Hu JL, Wu Y, Tang FY, Liu WD, Ding KQ, Zhao Y, Peng ZH, Yu RB, Wang H, Shen HB, Chen F (2012). Geographical information systems-based spatial analysis and implications for syphilis interventions in Jiangsu province, People's Republic of China. Geospat Health.

[186] Fitzpatrick G, Ward M, Ennis O, Johnson H, Cotter S, Carr MJ, O Riordan B, Waters A, Hassan J, Connell J, Hall W, Clarke A, Murphy H, Fitzgerald M (2012). Use of a geographic information system to map cases of measles in real-time during an outbreak in Dublin, Ireland, 2011. Euro Surveill.

[187] Ghetian CB, Parrott R, Volkman JE, Lengerich EJ (2008). Cancer registry policies in the United States and geographic information systems applications in comprehensive cancer control. Health Policy.

[188] Kho A, Johnston K, Wilson J, Wilson SJ (2006). Implementing an animated geographic information system to investigate factors associated with nosocomial infections: a novel approach. Am J Infect Control.

[189] Lerner EB, Fairbanks RJ, Shah MN (2005). Identification of out-of-hospital cardiac arrest clusters using a geographic information system. Acad Emerg Med.

[190] Li F, Harmer P, Cardinal BJ, Vongjaturapat N (2009). Built environment and changes in blood pressure in middle aged and older adults. Prev Med.

[191] Maheu-Giroux M, Casapía M, Soto-Calle VE, Ford LB, Buckeridge DL, Coomes OT, Gyorkos TW (2010). Risk of malaria transmission from fish ponds in the Peruvian Amazon. Acta Trop.

[192] Miranda ML, Alicia Overstreet Galeano M, Tassone E, Allen KD, Horner RD (2008). Spatial analysis of the etiology of amyotrophic lateral sclerosis among 1991 Gulf War veterans. Neurotoxicology.

[193] Namosha E, Mueller I, Kastens W, Kiele R, Kasehagen L, Siba PM (2010). Mapping the prevalence of malaria in rural Papua New Guinea using a geographic information system. P N G Med J.

[194] Nath MJ, Bora AK, Yadav K, Talukdar PK, Dhiman S, Baruah I, Singh L (2013). Prioritizing areas for malaria control using geographical information system in Sonitpur district, Assam, India. Public Health.

[195] Odero W, Rotich J, Yiannoutsos CT, Ouna T, Tierney WM (2007). Innovative approaches to application of information technology in disease surveillance and prevention in Western Kenya. J Biomed Inform.

[196] Pouliou T, Elliott SJ (2009). An exploratory spatial analysis of overweight and obesity in Canada. Prev Med.

[197] Shirayama Y, Phompida S, Shibuya K (2009). Geographic information system (GIS) maps and malaria control monitoring: intervention coverage and health outcome in distal villages of Khammouane province, Laos. Malar J.

[198] Springer YP, Samuel MC, Bolan G (2010). Socioeconomic gradients in sexually transmitted diseases: a geographic information system-based analysis of poverty, race/ethnicity, and gonorrhea rates in California, 2004-2006. Am J Public Health.

[199] Upadhyayula SM, Mutheneni SR, Kumaraswamy S, Kadiri MR, Pabbisetty SK, Yellepeddi VS (2012). Filaria monitoring visualization system: a geographical information system-based application to manage lymphatic filariasis in Andhra Pradesh, India. Vector Borne Zoonotic Dis.

[200] Wei L, Qian Q, Wang ZQ, Glass GE, Song SX, Zhang WY, Li XJ, Yang H, Wang XJ, Fang LQ, Cao WC (2011). Using geographic information system-based ecologic niche models to forecast the risk of hantavirus infection in Shandong Province, China. Am J Trop Med Hyg.

[201] Irene Wong YL, Stanhope V (2009). Conceptualizing community: a comparison of neighborhood characteristics of supportive housing for persons with psychiatric and developmental disabilities. Soc Sci Med.

[202] Yao J, Murray AT, Agadjanian V, Hayford SR (2012). Geographic influences on sexual and reproductive health service utilization in rural Mozambique. Appl Geogr.

[203] Burton NW, Haynes M, Wilson LA, Giles-Corti B, Oldenburg BF, Brown WJ, Giskes K, Turrell G (2009). HABITAT: a longitudinal multilevel study of physical activity change in mid-aged adults. BMC Public Health.

[204] Kruger DJ, Brady JS, Shirey LA (2008). Using GIS to facilitate community-based public health planning of diabetes intervention efforts. Health Promot Pract.

[205] Azzolini C (2011). A pilot teleconsultation network for retinal diseases in ophthalmology. J Telemed Telecare.

[206] Beebe LH, Smith K, Bennett C, Bentley K, Walters AB, Hancock B, Farmer SY, Earle K, White S (2010). Keeping in touch. Cell phone use in people with schizophrenia disorders. J Psychosoc Nurs Ment Health Serv.

[207] Bryen DN, Carey A, Friedman M (2007). Cell phone use by adults with intellectual disabilities. Intellect Dev Disabil.

[208] Buck DS, Rochon D, Turley JP (2005). Taking it to the streets: recording medical outreach data on personal digital assistants. Comput Inform Nurs.

[209] Grasso MA, Yen MJ, Mintz ML (2006). Survey of handheld computing among medical students. Comput Methods Programs Biomed.

[210] Guerrieri R, Kokinova M (2009). Does instruction in the use of personal digital assistants increase medical students' comfort and skill level?. Med Ref Serv Q.

[211] Khan AN, Frank J, Geria R, Davidson S (2007). Utilization of personal digital assistants (PDAS) by pediatric and emergency medicine residents. J Emerg Med.

[212] Kuiper R (2008). Use of personal digital assistants to support clinical reasoning in undergraduate baccalaureate nursing students. Comput Inform Nurs.

[213] Cornelius JB, St Lawrence JS, Howard JC, Shah D, Poka A, McDonald D, White AC (2012). Adolescents' perceptions of a mobile cell phone text messaging-enhanced intervention and development of a mobile cell phone-based HIV prevention intervention. J Spec Pediatr Nurs.

[214] Fischer HH, Moore SL, Ginosar D, Davidson AJ, Rice-Peterson CM, Durfee MJ, MacKenzie TD, Estacio RO, Steele AW (2012). Care by cell phone: text messaging for chronic disease management. Am J Manag Care.

[215] Lawler FH, Cacy J (2005). Utilization and value of personal digital assistants on an epidemiology final examination. Teach Learn Med.

[216] Lee S, Chib A, Kim JN (2011). Midwives' cell phone use and health knowledge in rural communities. J Health Commun.

[217] Leite L, Buresh M, Rios N, Conley A, Flys T, Page KR (2013). Cell phone utilization among foreign-born Latinos: a promising tool for dissemination of health and HIV information [epub ahead of print]. J Immigr Minor Health.

[218] McCord G, Pendleton BF, Schrop SL, Weiss L, Stockton L, Hamrich LM (2009). Assessing the impact on patient-physician interaction when physicians use personal digital assistants: a Northeastern Ohio Network (NEON) study. J Am Board Fam Med.

[219] Menachemi N, Perkins RM, van Durme DJ, Brooks RG (2006). Examining the adoption of electronic health records and personal digital assistants by family physicians in Florida. Inform Prim Care.

[220] Nguyen HQ, Gill DP, Wolpin S, Steele BG, Benditt JO (2009). Pilot study of a cell phone-based exercise persistence intervention post-rehabilitation for COPD. Int J Chron Obstruct Pulmon Dis.

[221] Person AK, Blain ML, Jiang H, Rasmussen PW, Stout JE (2011). Text messaging for enhancement of testing and treatment for tuberculosis, human immunodeficiency virus, and syphilis: a survey of attitudes toward cellular phones and healthcare. Telemed J E Health.

[222] Ranson SL, Boothby J, Mazmanian PE, Alvanzo A (2007). Use of personal digital assistants (PDAs) in reflection on learning and practice. J Contin Educ Health Prof.

[223] Rice E, Lee A, Taitt S (2011). Cell phone use among homeless youth: potential for new health interventions and research. J Urban Health.

[224] Rice E, Rhoades H, Winetrobe H, Sanchez M, Montoya J, Plant A, Kordic T (2012). Sexually explicit cell phone messaging associated with sexual risk among adolescents. Pediatrics.

[225] Smith T, Darling E, Searles B (2011). 2010 survey on cell phone use while performing cardiopulmonary bypass. Perfusion.

[226] Stroud SD, Erkel EA, Smith CA (2005). The use of personal digital assistants by nurse practitioner students and faculty. J Am Acad Nurse Pract.

[227] Tilghman J, Raley D, Conway JJ (2006). Family nurse practitioner students utilization of Personal Digital Assistants (PDAs): implications for practice. ABNF J.

[228] Turner PR, Burgin C, Funderburg KM, Van Grevenhof J, Knehans AW (2008). Use of personal digital assistants among dietitians and dietetic students in Oklahoma: should programs incorporate PDA training into their curricula?. J Allied Health.

[229] Cleland J, Caldow J, Ryan D (2007). A qualitative study of the attitudes of patients and staff to the use of mobile phone technology for recording and gathering asthma data. J Telemed Telecare.

[230] Donnelly MP, Nugent CD, Craig D, Passmore P, Mulvenna M (2008). Development of a cell phone-based video streaming system for persons with early stage Alzheimer's disease. Conf Proc IEEE Eng Med Biol Soc.

[231] Eze N, Lo S, Bray D, Toma AG (2005). The use of camera mobile phone to assess emergency ENT radiological investigations. Clin Otolaryngol.

[232] Franklin VL, Greene A, Waller A, Greene SA, Pagliari C (2008). Patients' engagement with "Sweet Talk" - a text messaging support system for young people with diabetes. J Med Internet Res.

[233] Elkaim M, Rogier A, Langlois J, Thevenin-Lemoine C, Abelin-Genevois K, Vialle R (2010). Teleconsultation using multimedia messaging service for management plan in pediatric orthopaedics: a pilot study. J Pediatr Orthop.

[234] Garrett BM, Jackson C (2006). A mobile clinical e-portfolio for nursing and medical students, using wireless personal digital assistants (PDAs). Nurse Educ Today.

[235] Garrett B, Klein G (2008). Value of wireless personal digital assistants for practice: perceptions of advanced practice nurses. J Clin Nurs.

[236] Momtahan KL, Burns CM, Sherrard H, Mesana T, Labinaz M (2007). Using personal digital assistants and patient care algorithms to improve access to cardiac care best practices. Stud Health Technol Inform.

[237] Salbach NM, Veinot P, Jaglal SB, Bayley M, Rolfe D (2011). From continuing education to personal digital assistants: what do physical therapists need to support evidence-based practice in stroke management?. J Eval Clin Pract.

[238] Hirano YO, Hirata N (2008). Cell phone e-mail as a means to collect information on pregnancy and delivery: a pilot study in Japan. Fukuoka Igaku Zasshi.

[239] Lasserre K, Eley D, Baker P, Kruesi L (2010). Medical students out of town but not out of touch: Use of personal digital assistants to improve access to clinical information and enhance learning at the point of care in rural and remote Australia. Aust J Rural Health.

[240] Lim EJ, Haar J, Morgan J (2008). Can text messaging results reduce time to treatment of Chlamydia trachomatis?. Sex Transm Infect.

[241] Joo NS, Kim BT (2007). Mobile phone short message service messaging for behaviour modification in a community-based weight control programme in Korea. J Telemed Telecare.

[242] Kim DK, Yoo SK, Kim SH (2005). Instant wireless transmission of radiological images using a personal digital assistant phone for emergency teleconsultation. J Telemed Telecare.

[243] Lim TH, Choi HJ, Kang BS (2010). Feasibility of dynamic cardiac ultrasound transmission via mobile phone for basic emergency teleconsultation. J Telemed Telecare.

[244] Park W, Lee HN, Jeong JS, Kwon JH, Lee GH, Kim KD (2012). Optimal protocol for teleconsultation with a cellular phone for dentoalveolar trauma: an in-vitro study. Imaging Sci Dent.

[245] Yoon KH, Kim HS (2008). A short message service by cellular phone in type 2 diabetic patients for 12 months. Diabetes Res Clin Pract.

[246] Lund S, Hemed M, Nielsen BB, Said A, Said K, Makungu MH, Rasch V (2012). Mobile phones as a health communication tool to improve skilled attendance at delivery in Zanzibar: a cluster-randomised controlled trial. BJOG.

[247] Chandhanayingyong C, Tangtrakulwanich B, Kiriratnikom T (2007). Teleconsultation for emergency orthopaedic patients using the multimedia messaging service via mobile phones. J Telemed Telecare.

[248] Curioso WH, Kurth AE, Cabello R, Segura P, Berry DL (2008). Usability evaluation of Personal Digital Assistants (PDAs) to support HIV treatment adherence and safer sex behavior in Peru. AMIA Annu Symp Proc.

[249] Lester RT, Ritvo P, Mills EJ, Kariri A, Karanja S, Chung MH, Jack W, Habyarimana J, Sadatsafavi M, Najafzadeh M, Marra CA, Estambale B, Ngugi E, Ball TB, Thabane L, Gelmon LJ, Kimani J, Ackers M, Plummer FA (2010). Effects of a mobile phone short message service on antiretroviral treatment adherence in Kenya (WelTel Kenya1): a randomised trial. Lancet.

[250] van der Kop ML, Karanja S, Thabane L, Marra C, Chung MH, Gelmon L, Kimani J, Lester RT (2012). In-depth analysis of patient-clinician cell phone communication during the WelTel Kenya1 antiretroviral adherence trial. PLoS One.

[251] Hsieh CH, Jeng SF, Chen CY, Yin JW, Yang JC, Tsai HH, Yeh MC (2005). Teleconsultation with the mobile camera-phone in remote evaluation of replantation potential. J Trauma.

[252] Hsieh JC, Lin BX, Wu FR, Chang PC, Tsuei YW, Yang CC (2010). Ambulance 12-lead electrocardiography transmission via cell phone technology to cardiologists. Telemed J E Health.

[253] Jotkowitz A, Oh J, Tu C, Elkin D, Pollack LA, Kerpen H (2006). The use of personal digital assistants among medical residents. Med Teach.

[254] Peleg R, Avdalimov A, Freud T (2011). Providing cell phone numbers and email addresses to Patients: the physician's perspective. BMC Res Notes.

[255] Peleg R, Nazarenko E (2012). Providing cell phone numbers and e-mail addresses to patients: The patient's perspective, a cross sectional study. Isr J Health Policy Res.

[256] Mitchell KJ, Bull S, Kiwanuka J, Ybarra ML (2011). Cell phone usage among adolescents in Uganda: acceptability for relaying health information. Health Educ Res.

[257] Siedner MJ, Haberer JE, Bwana MB, Ware NC, Bangsberg DR (2012). High acceptability for cell phone text messages to improve communication of laboratory results with HIV-infected patients in rural Uganda: a cross-sectional survey study. BMC Med Inform Decis Mak.

[258] Phabphal K, Hirunpatch S (2008). The effectiveness of low-cost teleconsultation for emergency head computer tomography in patients with suspected stroke. J Telemed Telecare.

[259] Saw S, Loh TP, Ang SB, Yip JW, Sethi SK (2011). Meeting regulatory requirements by the use of cell phone text message notification with autoescalation and loop closure for reporting of critical laboratory results. Am J Clin Pathol.

[260] Serdar MA, Turan M, Cihan M (2008). Rapid access to information resources in clinical biochemistry: medical applications of personal digital assistants (PDA). Clin Exp Med.

[261] Sharma R, Hebbal M, Ankola AV, Murugabupathy V (2011). Mobile-phone text messaging (SMS) for providing oral health education to mothers of preschool children in Belgaum City. J Telemed Telecare.

[262] Skinner D, Donald S, Rivette U, Ulrike R, Bloomberg C, Charissa B (2007). Evaluation of use of cellphones to aid compliance with drug therapy for HIV patients. AIDS Care.

[263] Bell BS, Hoskins RE, Pickle LW, Wartenberg D (2006). Current practices in spatial analysis of cancer data: mapping health statistics to inform policymakers and the public. Int J Health Geogr.

[264] Caley LM, Shiode N, Shelton JA (2008). Community/campus partnership: tailoring geographic information systems for perinatal health planning. Prog Community Health Partnersh.

[265] Kamadjeu R, Tolentino H (2006). Web-based public health geographic information systems for resources-constrained environment using scalable vector graphics technology: a proof of concept applied to the expanded program on immunization data. Int J Health Geogr.

[266] Parrott R, Hopfer S, Ghetian C, Lengerich E (2007). Mapping as a visual health communication tool: promises and dilemmas. Health Commun.

[267] Parrott R, Volkman JE, Lengerich E, Ghetian CB, Chadwick AE, Hopfer S (2010). Using geographic information systems to promote community involvement in comprehensive cancer control. Health Commun.

[268] Joyce K (2009). "To me it's just another tool to help understand the evidence": public health decision-makers' perceptions of the value of geographical information systems (GIS). Health Place.

[269] Koenig A, Samarasundera E, Cheng T (2011). Interactive map communication: pilot study of the visual perceptions and preferences of public health practitioners. Public Health.

[270] Farrell MJ, Rose L (2008). Use of mobile handheld computers in clinical nursing education. J Nurs Educ.

[271] Goldsworthy S, Lawrence N, Goodman W (2006). The use of personal digital assistants at the point of care in an undergraduate nursing program. Comput Inform Nurs.

[272] Mcleod TG, McNaughton DA, Hanson GJ, Cha SS (2009). Educational effectiveness of a personal digital assistant-based geriatric assessment tool. Med Teach.

[273] Strayer SM, Pelletier SL, Martindale JR, Rais S, Powell J, Schorling JB (2010). A PDA-based counseling tool for improving medical student smoking cessation counseling. Fam Med.

[274] Tempelhof MW, Garman KS, Langman MK, Adams MB (2009). Leveraging time and learning style, iPod vs. realtime attendance at a series of medicine residents conferences: a randomised controlled trial. Inform Prim Care.

