# Supplementary material for: Mobile Technologies and Geographic Information Systems to Improve Health Care Systems: A Literature Review
Source: JMIR Mhealth Uhealth. 2014 May 8;2(2):e21. doi: 10.2196/mhealth.3216 (PMC4114429; doi:10.2196/mhealth.3216)
Supplement: Supplementary file 1 [file mhealth_v2i2e21_app1.pdf]

Multimedia Appendix 1. Treatment and disease management – Overview of papers mHealth articles

Research Country and reference	Sample size	Device	Aims	Key effects
USA [1]	8	Voice	To evaluate the practicality and acceptability of using cell phone reminders to improve HAART adherence in HIV-infected adolescents	Cell phone reminders were practical and acceptable to pilot study participants. A 12-week intervention was not adequate for most subjects.
Ireland [2]		SMS	To audit non-attendance rates at ENT out-patient clinics following the introduction of a text reminder system.	Sending text message reminders is a simple and cost-effective way to improve non-attendance at ENT outpatient clinics
Kenya [3]		SMS	To compare the effectiveness of cell phone-supported SMS to standard care on adherence, quality of life, retention, and mortality in a population receiving ART.	No findings
UK [5]		SMS	To evaluate the use of SMS to reduce nonattendance at out-patient clinics	The impact is modest, but so also is the cost, about 7.50 pounds per "no-show" avoided.
Denmark [6]	26	SMS	To examine the impact of receiving a daily text message reminder on one’s cell phone on adherence to asthma treatment.	Daily SMS reminders improved anti-asthmatic medication; the perception of receiving a daily SMS was positive.

Peru [7]		SMS	To characterize effective ART reminders strategies for people living with HIV/AIDS to improve adherence using SMS based on patient perspectives.	The participants preferred text messages over recorded voice messages or phone calls as reminder alerts because they are easier, more confidential, and more readily-available; overall participants were in favour of receiving medication reminders on their cell phones, and preferred characteristic for the reminders was that they be motivational.
Peru [8]		SMS, voice and PDAs	To report on perceptions towards use of ICT as a means to support antiretroviral medication adherence and HIV transmission risk reduction.	Patients had positive perceptions about using the Internet, cell phones and PDAs for HIV health promotion interventions.
Spain [9]	254	SMS	To assess the impact of a SMS-based strategy on adherence to antipsychotic treatment.	Daily treatment SMS reminders are efficacious in the enhancement of adherence to oral antipsycothic treatment.
France [10]	424	SMS	To assess the feasibility and acceptability of sending a daily SMS reminder to remind soldiers to take their malaria chemoprophylaxi, and to assess the impact of the daily reminder SMS on	SMS did not increase malaria chemoprophylaxis compliance above baseline. The intervention was feasible and SMS reminders were very unseful.

			chemoprophylaxis compliance	
Ireland [11]	59	SMS	To assess the levels of self-reported adherence to antidepressant medication in the community and to investigate the acceptability of text message reminders to self-reported unintentional non-adherers	SMS reminders were acceptable to approximately 60% of patients who reported being unintentionally non-adherent.
Netherlands [12]	104	SMS	To investigate the effect of these SMS reminders on adherence to oral antidiabetics in patients using RTMM and investigate patients' experiences with RTMM	RTMM with SMS reminders improves adherence of type 2 diabetes patients, especially the precision with which patients follow their prescribed regimen, and is well accepted by patients.
Netherlands [13]		SMS	To describe the design of an intervention study aimed at evaluating the effect of RTMM on adherence to oral antidiabetics.	No findings
Korea [14]	69	Mobile phone	To compare the effect of a mobile phone with a glucometer integrated into the battery pack on management of type 2 diabetes to the Internet-based glucose monitoring system.	Two-way communication between doctors and patients using the diabetes phone was as effective for glucose control as the previously-studied Internet-based monitoring system and it was good for patient satisfaction and adherence.
USA [16]		SMS	To (1) develop a diabetes-	Participants expressed high

			focused text message-based reminder and data collection system in an urban African-American population; (2) assess the feasibility of its implementation; and (3) assess logistical challenges and perceived benefits of such a system on diabetes self-management.	satisfaction with text messages; text messaging may be a feasible and useful approach to improve diabetes self-management in urban African Americans.
USA [17]	30	SMS	To examine the feasibility of utilizing of cell phone technology to assist with diabetes self-care in a clinic population as well as its impact on clinical outcomes.	The intervention had a positive impact on some clinical outcome and self-efficacy; it was feasible.
Korea [18]	33	SMS	To investigate the effect of a nurse short message service by cellular phone and the Internet on glycosylated hemoglobin (HbA1c) levels and adherence to diabetes control recommendations.	After 12 weeks, the patients had a mean decrease of 1.1% in HbA1c level and an increase in diabetic medication taking, 30 minutes of physical exercise, and foot care adherence.
Austria [19]	10	Mobile phone	To evaluate the feasibility and user acceptance of a mobile phone–based data service to assist DM1 patients on intensive insulin treatment.	Participants' attitudes were positive; mobile phone provided an ubiquitous, easy-to-use, and cost efficient solution for patient-centered data acquisition in the management of DM1.
Canada [20]		Mobile	To develop and pilot-test a	Patients' acceptance rate

		phone	home blood-pressure (BP) tele-management system that actively engages patients in the process of care.	was high. Home BP showed a significant improvement during the intervention.
Austria [21]	25	Mobile phone	To assess the technical feasibility, usability and acceptance of a web-based therapy management system with mobile phone access to support obese patients.	Participants' attitudes were positive. Mobile phones can be utilized as patient terminals for therapy in patients suffering from obesity.
USA [22]	36	Mobile phone	To compare active interventions and usual care for glucose control in a randomized clinical trial (RCT) among persons with diabetes cared for by primary care physicians (PCPs) over the course of 1 year.	No findings
USA [23]	163	Mobile phone	To test whether adding mobile application coaching and patient/ provider web portals to community primary care compared with standard diabetes management would reduce glycated hemoglobin levels in patients with type 2 diabetes.	Mobile phone-based treatment/ behavioral coaching intervention improved glycated hemoglobin.
USA [24]	4	SMS	To test the feasibility and utilization of tracking asthma symptoms through	This method of management is feasible; participants' satisfaction was good.

			an innovative mobile phone application.	
UK [25]	48	Mobile phone	To explore the opinions and concerns of people with asthma and primary care clinicians on the potential role of mobile phone monitoring technology in supporting asthma self-management.	Participant’s considered the intervention as facilitating guided self-management, and provided much-needed confidence.
UK [26]	46	PDA	To conduct an observational study using electronic peak flow monitoring and mobile phone technology in a UK general practice population.	The intervention increased awareness and information about asthma, improved ability to monitor/manage the condition with the feedback.
USA [27]	30	Mobile phone	To (1) assess the impact on A1c of a cell phone-based diabetes management software system used with web-based data analytics and therapy optimization tools; (2) examine health care provider (HCP) adherence to prescribing guidelines and assessed HCPs' adoption of the technology.	Participants achieved statistically significant improvements in A1c. HCP and patient satisfaction with the system was clinically and statistically significant.
USA [28]	40	SMS	To determine if daily automated SMS would result in increased adherence to	Electronic reminders in the form of daily, customized SMS were not associated with significant differences

			recommended use of topical acne medication and consequently greater improvement in acne.	in adherence to topical medications in patients with mild to moderate acne and had no significant effect on therapeutic response.
USA [29]	48	SMS	To evaluate the feasibility, acceptability, and preliminary efficacy of text message reminders to improve adherence to ART for youth living with HIV/AIDS.	Personalized, interactive, da ily SMS reminders were feasible and acceptable, and they significantly improved self-reported adherence.
USA [30]	16	SMS	To assess the feasibility of Ecological Momentary Assessment via text messaging to measure asthma medication use and symptoms in African Americans.	The assessment found low medication adherence rates and reasons for missing medication consisten t with previous research with youth with asthma.
USA [31]	580	SMS	To evaluate medication adherence among patients opting to receive text message medication reminders and a well-matched control cohort.	The SMS intervention improved medication adherence, and assisted in preserving higher rates of adherence over time.
Korea [32]		mobile phone	To investigate the efficacy of a u-healthcare service using Zigbee and mobile phone for elderly patients with diabetes mellitus or heart diseases.	Patients' satisfaction was relatively high; The mean transmission frequencies were 2.1 times/day in blood glucose monitoring and 6.1 times/day in ECG.
Canada [33]	102	SMS	To determine the effectiveness of cell phone wireless text messaging for improving adherence	Wireless text messaging reminders improved adherence to healthy behaviour (taking vitamin C

			to a healthy behaviour.	for preventive reasons) over a 1-month intervention
Kenya [34]	431	SMS	To test the efficacy of SMS reminders on adheren ce to ART among patients attending a rural clinic	Participants receiving SMS reminders achieved high adherence rate and were significantly less likely to experience treatment interru ptions exceeding 48 h during the 48-week follow-up period than participants in the control group.
Cameroon [35]	200	SMS	To investigate the use of motivational SMS to improve adherence to ART over six months.	Standardized motivational SMS did not significantly improve adherence to ART.
Brazil [36]	21	SMS	To assess whether a warning system based on mobile SMS increases the adherence of HIV-infected Brazilian women to antiretroviral drug-based treatment regimens and their impressions and satisfaction with respect to incoming messages.	SMS messaging can help Brazilian women living with HIV/AIDS to adhere to antiretroviral therapy for a period of at least 4 months.
New Zealand [37]	260	SMS	To investigate whether a text message programme targeted at changing patients' illness and medication beliefs would improve adherence in young adult asthma patients.	The intervention increased adherence to asthma preventer inhaler and may be useful for other illnesses where adherence is a major issue.
UK [38]		SMS	To audit non-attendance rates at ENT out-patient	The mean rate of non-attendance reduced from

			clinics following the introduction of a text reminder system.	33.6 to 22 per cent. A text message reminder is a simple and cost-effective way to improve non-attendance at ENT out-patient clinics.
