# Supplementary material for: Mobile Technologies and Geographic Information Systems to Improve Health Care Systems: A Literature Review
Source: JMIR Mhealth Uhealth. 2014 May 8;2(2):e21. doi: 10.2196/mhealth.3216 (PMC4114429; doi:10.2196/mhealth.3216)
Supplement: Supplementary file 2 [file mhealth_v2i2e21_app2.pdf]

Multimedia Appendix 2. Data collection and disease surveillance – overview of mHealth articles

Research Country and reference	Sample size	Device	Aims	Findings
Sweden [39]		SMS; Telephone	To compare the use of SMS on mobile phones and the use of telephone interviews in collecting self-reported data about influenza vaccination.	SMS is a feasible method for collection of information on vaccination status data among the Swedish population compared to telephone interviews
Norway [40]		PDA	To assess how PDAs perform as collection tools of patient-reported outcomes in clinical research compared to pen and paper diaries in terms of feasibility, protocol compliance, data accuracy, and subject acceptability.	The PDA method seems to perform better than P&P in most of the selected outcomes. Technical malfunction is the chief disadvantage with the PDA method.
Norway [41]		PDA	To compare daily and weekly registrations of self-reported health status measures between PDA and paper-pencil (PP) format regarding scores, variation, and feasibility.	Daily assessments with PDA may be efficiently used for frequent data collection because this format performs similarly to the traditional PP format.
USA [42]	10	PDA	To determine the feasibility of using PDA-based technology for tracking and analysis of food intake in low-income pregnant women.	PDA-based software may be an acceptable method for obtaining food records to provide more accurate assessments of dietary intake in this vulnerable population.

USA [43]	19	PDA	To compare the completeness of data collection using paper forms and using electronic forms loaded on PDAs.	PDA produced more complete data than the paper method or electronic forms.
USA [44]		PDA	To describe the use of PDAs by doctor of pharmacy (PharmD) students; determine the reliability of psychometric constructs that determine technology acceptance; and determine constructs that directly correlate with PDA use.	The majority of pharmacy students used their PDAs at least weekly and find them most useful for looking up drug information.
USA [45]	63	SMS	To determine the feasibility and acceptability of using two-way SMS texts to collect situati onal assessment (SA) data in simulated disaster events.	Using two-way SMS communication for surveillance and reporting was feasible among a group of motivated students. Similar methods may provide timely data during public health critical events.
Germany [46]		PDA	To investigate the effectiveness of PDA-based electronic record system (Clinic Coach(c)) to clinical routine	The combination of PDAs and ClinicCoach(c) is a reliable and to clinical routine well adapted system that allows digital documentation at the bedside.
Canada [47]	20	PDA	To evaluate (1) the ability of pediatricians to use PDAs to capture patient care data; (2) the feasibility of faculty	Participants accepted the PDA method of data capture. The method added significant information in capturing activities.

			documentation of clinical and educational workload data.	
Canada [48]		PDA	To describe a scalable, multiuser, PDA-based tool for pharmacist collection of data on DRPs.	A PDA-based documentation tool was successfully used in a multisite health care organization to collect data on DRPs and document pharmacist interventions.
UK [49]		SMS	To test the reliability, validity, acceptability, and practicality of short message service (SMS) messaging for collection of research data.	SMS was a reliable and valid method for capturing research data.
UK [50]	37	SMS	To compare the use of 'text messaging' and computer-based interviewing (CBI) with the standard diary method.	SMS and CBI are alternatives to written diaries as methods of data collection; both have the advantage of daily reminders, rapid response and quick data analysis. SMS was a preferred method over the written diary.
Austria [51]	20	Mobile phone	To test the reliability, acceptability and feasibility of a home-monitoring system for cardiac patients.	The web-based home-monitoring system was reliable and easy to handle for both patients and health care professionals.
Japan [52]	38	PDA	To evaluate a PDA with camera and mobile telephone card, called Wellnavi, for dietary	This study suggests that a PDA may be a valid and convenient instrument for evaluating dietary intake.

			intake.	
Japan [53]	27	PDA	To evaluate a PDA with camera and mobile telephone card (Wellnavi) for dietary intake.	A hand-held PDA may be a valid and convenient instrument for evaluating dietary intake.
Ghana [54]		SMS	To evaluate the use of cell phones by professional and traditional birth attendants in rural Africa for reporting postpartum hemorrhage (PPH) data.	Reporting from rural-based providers may present a more accurate picture of what occurs in remote communities because it happens in real time.
Kenya [55]		PDA	To evaluate the acceptability, data quality and usefulness of PDAs.	PDAs had high rates of missing data but helped identify clinics that were under testing for HIV or under prescribing CTX.
Kenya [56]		PDA	To assess the strengths and weaknesses of different data collection methodologies: paper questionnaires and PDAs.	PDAs may be successfully used in implementing surveys in the African context. Use of PDAs speeds data collection, improves completeness and, most importantly, increases data quality.
Kenya [57]	433	PDA	To describe the use of an existing electronic medical record and PDAs to assess the care and outcomes of one of the most prevalent conditions at the center: acute respiratory tract infections.	EMRs and PDA are useful tools for performing prospective clinical research in resource constrained developing countries.
Peru [58]		PDA	To evaluate the quality of sexual behavior data collected with handheld	It is feasible to develop a low-cost application for handheld computers, and

			computers in comparison with paper-based questionnaires.	that handheld computers are feasible alternatives for collecting field data in a developing country.
Peru [59]		PDA	To assess the data collection efficiency of each system and the resources required to develop, implement and transfer the PDA-based system to a resource- poor setting.	A PDA-based system drastically reduced the effort required to collect TB laboratory results from remote locations.
Peru [60]		PDA	To evaluate the effectiveness of a PDA-based system for collecting tuberculosis test results and to compare this new system to the previous paper-based system.	The PDA-based system had a significant effect on processing times and errors. The intervention reduced the work-hours necessary to process results by 70% and was preferred by all users.
Angola [61]	10	PDA	To explore the acceptability of handheld computers for HIV/AIDS data collection and to identify potential barriers to acceptance	Personal and cultural beliefs influence participant acceptance of handheld computers in Angola.
Swaziland [62]		SMS	To report results after implementation of the LabPush system designed to test whether text-message delivery of laboratory results to mobile phone of the head nurse of the clinics could shorten turnaround time (TAT) significantly.	SMS reporting of critical lab results to clinics has improved TAT of the national laboratory in Swaziland. Missing rates of test results were reduced significantly.

Malaysia [63]		SMS	To evaluate the effectiveness of using text messaging as a means to collect data for a medical research project.	SMS is an excellent instrument for collecting weekly symptom reports in response to trial medication, reminding trial subjects to attend face to face visits and completing more complex paper based evaluation.
India [64]		Internet SMS, voice	To provide a quantitative evaluation of data entry accuracy using low-cost mobile phones in a resource- constrained environment.	The error rates for electronic forms and SMS may be too high to deploy these solutions in a critical application. In contrast, the accuracy of the voice interface was an order of magnitude better.
India [65]		Mobile phone	To investigate the effectiveness and efficiency gains in collect outpatient health information from primary health centres and health sub centres through mobile applications for detecting disease outbreaks in near-real-time.	The intervention improved timeliness for real-time detection of any disease outbreak Health workers were able to submit reliable patient health data using mobile phone, which makes it a friendly, efficient, and cost effective tool of data collection.
Iran [66]		SMS	To introduce cell phone surveillance (CPS) system for surveillance of communicable diseases in countries with limited resources.	CPS reduced time for reporting, seemed to be fast and cheaper than traditional way, as well as having a grate rate of reliability.
China [67]		Mobile phone	To describe the utilization of mobile phones infectious disease	The mobile phone is a useful communication tool for infectious disease

			surveillance after the catastrophic earthquake.	surveillance in areas hit by natural disasters
