# Supplementary material for: Mobile Technologies and Geographic Information Systems to Improve Health Care Systems: A Literature Review
Source: JMIR Mhealth Uhealth. 2014 May 8;2(2):e21. doi: 10.2196/mhealth.3216 (PMC4114429; doi:10.2196/mhealth.3216)
Supplement: Supplementary file 3 [file mhealth_v2i2e21_app3.pdf]

Multimedia Appendix 3. Disease surveillance – overview of GIS articles

Research Country and reference	Aims	Findings
Canada [69]	To outline the context, architecture and different capabilities of the ISPHM-WNV, including its spatial and cartographic functionalities.	The ISPHM-WNV facilitates the collection, localization, management and analysis of monitoring data; it also allows for the display of the results of analyses on maps, tables and statistical diagrams.
France [70]	To develop user-friendly interfaces for remote data entry and GIS tools providing real-time atlas of the epidemiologic situation in any location.	No findings
USA [71]	To assess whether the diagnoses of respiratory and GI illnesses as defined by visits to primary care offices and an emergency department setting in a defined geographic community, follow a spatial pattern as the illnesses cycle through the community over time.	GIS is feasible to assess visit rates for common illnesses in a defined community and identified spatial variability over time.
USA [72]	To conducted sentinel surveillance among women entering the jail system of San Francisco from 1999 to 2001 to track trends in HIV incidence, HIV preva lence, and related risk behaviour.	Maps showed that the communities in which arrested women reside are also those with the highest concentrations of newly detected female HIV cases, AIDS cases, and clients of substance use programs. The combined strategy of using sentinel surveillance in the jail setting and GIS to map the spatial distribution of disease provides a useful tool to identify patterns of risk in hard-to-reach,

		vulnerable populations of women.
Netherlands [73]	To investigate whether it would be possible to develop a GIS for basic infrastructure planning and management at local level.	Spatial information is an identified need at local level. Open source GIS software can be used to develop a system to provide local-level stakeholders with spatial information.
South Africa [74]	To assess the utility and feasibility of using free (non-licensed), and easy-to-use Social Web and GeoWeb tools for injury surveillance in low- resource settings.	This study illustrates the great potential for these technologies to be leveraged for public health surveillance in resource-constrained environments, given their ease-of-use and low-cost, and the sharing and collaboration capabilities they afford.
Nicaragua [75]	To describe the development of a low-cost mapping and georeferencing system which does not rely on continuous access to Internet.	This dengue surveillance program allows public health workers in resource-limited settings to accurately identify areas with high indices of mosquito infestation and interpret the spatial relationship of these areas with potential larval development sites such as garbage piles and large pools of standing water.
India [76]	To link and understand the health scenario in a GIS context with emphasis on HIV/AIDS.	Overall scenario of the spread of HIV/AIDS around the world is presented along with the Indian perspective.
Vanuatu [77]	To examine modern approaches to GR to define the spatial distribution of target populations to support contemporary malaria elimination i nterventions.	GR implemented using modern techniques has provided an effective and efficient operational tool for rapidly defining the spatial distribution of target populations in designated malaria elimination zones.
Congo [78]	To examine the individual and community-level factors that	The prevalence of HIV within 25 km of an individual's community is an

	increase an individual's risk for HIV infection in the Democratic Republic of Congo.	important positive indicator of HIV infection. Distance from a city is negatively associated with HIV infection overall and for women in particular.
Trinidad and Tobago [79]	To analyze the rabies epidemic of 1929 to 1937 in Trinidad from a geographical perspective, using GIS software as an analytical tool	The pattern of spread appears to be spatially linked to land use/land cover. the spatial of distribution of the disease followed a distinct pathway possible due to the use of electromagnetic capabilities of bats.
Australia [80]	To examine the application of a GIS-based spatial decision support system (SDSS) to automatically locate and map the distribution of confirmed malaria cases, rapidly classify active transmission foci, and guide targeted responses in elimination zones.	Supported key elements of surveillance-response including understanding epidemiological variation within target areas, implementing appropriate foci-specific targeted response, and consideration of logistical constraints and costs.
