# Supplementary material for: Mobile Technologies and Geographic Information Systems to Improve Health Care Systems: A Literature Review
Source: JMIR Mhealth Uhealth. 2014 May 8;2(2):e21. doi: 10.2196/mhealth.3216 (PMC4114429; doi:10.2196/mhealth.3216)
Supplement: Supplementary file 4 [file mhealth_v2i2e21_app4.pdf]

**Multimedia Appendix 4. Health support systems – overview of mHealth articles**

| Research Country and reference | Sample size | Device | Aims | Findings |
|---|---|---|---|---|
| Norway [81] | 9 | Mobile phone | To design a mobile dietary management support technologies for people with diabetes. | The system was ease of use and acceptable to patients. |
| Norway [82] | 290 | Internet; SMS, voice | To describe the rationale for the design of happy ending (HE), to assess the 12-month efficacy of HE in a sample of smokers willing to attempt to quit without the use of nicotine replacement therapy, and to explore the potential effect of HE on coping planning and self-efficacy (prior to quitting) and whether coping planning and self-efficacy mediate treatment effect. | This 12-month trial documents a long-term treatment effect of a fully automated smoking cessation intervention without the use of nicotine replacement therapy. The study adds to the promise of using digital media in supporting behavior change. |
| France [83] | 15 | Internet; SMS | To assess metabolic efficacy, safety and quality of life using Web and the cellular phone network for retrospective data transmission and SMS. | Long-term telemedical follow-up of insulin pump-treated patients using a cellular phone-, SMS- and Web-based platform is feasible, safe, does not alter quality of life and associated with a trend toward improved metabolic control. |
| UK [84] | 51 | PDA | To evaluate the effects of using an audio-visual | PDA-based videos are an acceptable approach for |

| | | | | |
|---|---|---|---|---|
| | | | animation (i.e., digital video) displayed on a PDA for patient education in a clinical setting. | teaching patients about health care. |
| UK [85] | 92 | SMS | To assess Sweet Talk, a text-messaging support system designed to enhance self-efficacy, facilitate uptake of intensive insulin therapy and improve glycaemic control in paediatric patients with Type 1 diabetes. | Sweet Talk was associated with improved self-efficacy and adherence. Sweet Talk may be an effective means of providing support. |
| UK [86] | 200 | Mobile phone | To conduct a pilot randomised controlled trial of mobile phone-based smoking cessation support intervention for the UK population. | Mobile phone-based smoking cessation is an innovative means of delivering smoking cessation support, which doubles the self-reported quit rate in the short term. |
| UK [87] | 5800 | SMS | To assess the effect of an automated smoking cessation programme delivered via mobile phone text messaging on continuous abstinence | The txt2stop smoking cessat ion programme significantly improved smoking cessation rates at 6 months and should be considered for inclusion in smoking cessation services. |
| Spain [88] | | Mobile phone | To describe the design of a connectivity interface based on the HL7 standard that allows the MOTOHEALTH mobile health care solution to communicate with external electronic healthcare record systems supporting HL7. | No findings |
| USA [89] | | SMS | To develop and test | The system is able to |

| | | | | |
|---|---|---|---|---|
| | | | a staff recall system, based in anesthesia information management system (AIMS), using SMS. | rapidly mobilize sufficient numbers of anesthesia personnel in response to an MCI, but actual performance cannot be predicted with confidence. The AIMS was simple, inexpensive, and easy to implement. |
| USA [90] | 40 | SMS, e-mail | To test the feasibility of implementing a fully automated, two-way text messaging system to encourage increased blood glucose (BG) using the Computerized Automated Reminder Diabetes System (CARDS). | Cell phone text messaging to promote BG monitoring is a viable and acceptable option in adolescents and young adults with diabetes. |
| USA [91] | 75 | SMS | To describe the development and evaluation of a text message–based intervention designed to help individuals lose or maintain weight over 4 months. | Text messages might prove to be a productive channel of communication to promote behaviors that support weight loss in overweight adults. |
| USA [92] | 31 | SMS | To describe the development a smoking cessation program using mobile phone text messaging to provide tailored and stage-specific messages to college smokers. | Mobile phone text messaging is a potentially efficacious and easily disseminated method for providing cessation interventions to young adult smokers. |
| USA [93] | 151 | PDA | To describe the: (a) manner in which PDA-based self-monitoring is integrated within the SCT- | PDA-based dietary monitoring is perceived by participants to be useful and acceptable. The approach |

| | | | | |
|---|---|---|---|---|
| | | | based intervention, (b) feasibility and acceptability of PDA-based dietary self-monitoring, and (c) issues encountered in teaching participants to self-monitor using a PDA. | used to instruct participants in use of the PDA and lessons learned are discussed. PDA technology shows promise as a tool for assisting those with type 2 diabetes in their efforts to manage their disease. |
| USA [94] | | SMS | To discuss the protocol decisions and content development for SMS Turkey, a smoking cessation text messaging program for adult smokers in Turkey. | The theory and protocols underpinning telephone-based smoking cessation programs were useful guides for developing similar SMS Turkey program components. |
| USA [95] | 50 | SMS | To develop, implement, and test a tailored short message service-based intervention for HIV-positive men who have sex with men. | Results confirm the feasibility of a tailored, SMS-based intervention designed to provide on-going behavioural reinforcement for HIV-positive men who have sex with men. |
| UK [96] | | SMS | To evaluate smokers' preferences for the Quittext program, which examines the feasibility and acceptability of delivering tailored smoking cessation advice via mobile phone text messaging. | Delivering quitting advice by text message was considered feasible and acceptable. |
| UK [97] | | SMS | To describe a participatory design (PD) methodology to develop a text message scheduling system for | The PD approach yielded a reliable, functional, acceptable and usable scheduling system to deliver |

| | | | | |
|---|---|---|---|---|
| | | | supporting young people with diabetes. | automated text messaging support to young people with diabetes. The longer-term usability, effectiveness and cost efficiency of the system have been successfully demonstrated in a randomized controlled trial. |
| Sweden [98] | 15 | PDA | To evaluate nurses' experiences of using a MDSS (LIFe-reader) in a PDA with a barcode reader, in order to obtain profiles of the patients' medication, regarding drug–drug interactions, therapeutic duplications, and warnings for drugs unsuitable for elderly in home care. | The LIFe-reader® has the potential to be a useful and user-friendly MDSS for nurses in home care when obtaining profiles of the patients' medication regarding drug–drug interactions, therapeutic duplications and warnings for drugs unsuitable for elderly. |
| Greece [99] | | Mobile phone | To present the design and development of a mobile phone application for Type 1 Diabetes Mellitus (T1DM) self-management. | The use of the mobile phone technologies along with data analysis methods might improve the self-management of T1DM. |
| Netherlands [100] | 210 | SMS | To evaluate the implementation of the Dutch guideline 'Pain in patients with cancer' to improve pain reporting, pain measurement and adequate pain therapy. | SMS alerts may serve as a tool to support self-management of patients. Therefore, the SMS-IVR intervention may increase the feeling of having control over one's life. |
| Italy [101] | 5 | SMS | To describe a fully automatic platform to transmit, using the SMS service, medical | The patients' compliance with the system was high. The web-based home- |

| | | | | |
|---|---|---|---|---|
| | | | data collected at home. | monitoring system was reliable and easy handle for health care professionals. |
| New Zealand [102] | 226 | MMS | To assess the effectiveness of a multimedia mobile phone intervention for smoking cessation. | The study was not able to demonstrate a statistically significant effect of the complex video messaging intervention compared with simple general health video messages. Participant's feedback was positive. |
| New Zealand [103] | 180 | Mobile phone | To develop and pilot test a youth-oriented multimedia smoking cessation intervention delivered solely by mobile phone. | The intervention is technically feasible, and the content developed was appropriate for this medium and is acceptable to target population. |
| New Zealand [104] | 1348 | SMS | To develop and test the novel mobile phone delivery of a depression prevention intervention for adolescents. | Key messages from CBT can be delivered by mobile phone, and young people report that these are helpful |
| Saudi Arabia [105] | 16 | SMS | To measure the effect of integrating SMS reminders with an electronic medical record (EMR) system on non-attendance rates in outpatient clinics in a Saudi hospital. | The integration showed a positive effect on the reduction of the non-attended appointments. |
| Tanzania [106] | | SMS | To demonstrate (1) that visibility of weekly stock levels of key anti-malarial medicines at the health facility level will promote action to eliminate and/or reduce stock-outs; (2) that a state-of-the-art data gathering | The SMS for Life pilot provided visibility of anti-malarial stock levels to support more efficient stock management using simple and widely available SMS technology, via a public-private partnership model |

| | | | | |
|---|---|---|---|---|
| | | | infrastructure can be made available via simple tools such as SMS and mobile telephones in remote locations in sub-Saharan Africa (3) the effectiveness of a public-private partnership model. | that worked highly effectively. |
| Tanzania [107] | | PDA | To report on research into the implementation and evaluation of a wireless hand-held clinical care management system. | The system was beneficial to the end-users and to the facility as a whole. |
| Peru [108] | | PDA | To describe the development and implementation of a PDA- based electronic system to collect, verify and upload monthly bacteriology data into the PIH-EMR. | The system significantly decrease delays in processing and errors with a positive user experience. |
| Peru [109] | | SMS, voice, email | To develop an interactive-computer system using cell phones and the Internet for real-time collection and transmission of adverse events related with metronidazole administration as presumptive treatment for vaginosis among female sex workers in Peru. | It is feasible to develop a public-health-surveillance system based on cell phones to collect data in real- time in Peru. |
| Peru, Kenya [110] | | PDA | To develop a Web-based application delivered on PDAs (Colecta-PALM in Peru, Pambazuko-PALM in Kenya), to collect data from HIV patients and to facilitate | PDAs may be a culturally appropriate way to support ART adherence and safer sex for PLHA. |

| | | | | |
|---|---|---|---|---|
| | | | HIV provider training. | |
| Korea [111] | | PDA | To present a prototype mobile clinical information system, MobileMed, which integrates the distributed and fragmented patient data across heterogeneous sources and makes them available through PDAs. | The PDA-based system support fast and efficient data communication regardless of the transaction frequencies. |
| South Africa [112] | | PDA/GPS | To assess the feasibility of using a PDA programmed with customised software and linked to a GPS receiver, to assist TB control programmes to trace patients who interrupt treatment in areas without useful street maps. | It is feasible to use a simple PDA/GPS device to locate the homes of patients. In densely populated informal settlements, GPS technology is more accurate than aerial photos in identifying homes and more efficient than addresses provided by participants. |
| Uganda [113] | | mobile phone | To detail the rapid design and testing of a pilot implementation of a mobile and web-based system for processing claims forms, based on two prior field visits to Uganda. | The choice of the mobile phone as a platform was affirmed by the health clinics, for reasons of battery life, design for readability, portability (susceptibility to theft), and ease of data entry. |
| China [114] | 100 | SMS | To present the develop a mobile pharmacy service system (MPSS) to deliver individualized pharmaceutical care via the SMS, with the aim of improving medication compliance and safety | Most patients were satisfied with the pharmaceutical care provided by SMS and they had positive attitudes to it. The SMS provided patients with rapid, effective medication guidance and pharmaceutical care after |

| | | | | |
|---|---|---|---|---|
| | | | | discharge. |
| China [115] | | PDA | To develop and trial a PDA-based data collection/entry system, and evaluate whether such a system could increase efficiency and reduce data transcription errors for public surveillance data collection in developing countries. | PDA-based data entry reduced or eliminated data entry errors; performed better in timeliness of receipt and data handling than paper and pencil; majority of participants preferred this method; provides a cost-effective alternative to the paper-based. |
| Trinidad and Tobago [116] | | Mobile phone | To describes MediNet, a mobile healthcare system that is being developed to personalize the self-care process for patients with both diabetes and cardiovascular disease | No findings |
| Kenya [117] | | PDA | To describe the development, cost effectiveness and implementation of a PDA based electronic system to collect, verify and manage data from a multi-site study on HIV/AIDS stigma and pregnancy in a rural, resource-poor area. | It is feasible to utilize PDAs for data collection in a multi-site observational study on HIV/AIDS stigma conducted in remote rural health facilities in Kenya. |
| India [118] | 322 | SMS, voice | To present important considerations in the design of a mobile phone-based adherence intervention in India. | Mobile phone-based intervention to influence adherence among HIV patients in our context. |
