# Supplementary material for: Mobile Technologies and Geographic Information Systems to Improve Health Care Systems: A Literature Review
Source: JMIR Mhealth Uhealth. 2014 May 8;2(2):e21. doi: 10.2196/mhealth.3216 (PMC4114429; doi:10.2196/mhealth.3216)
Supplement: Supplementary file 5 [file mhealth_v2i2e21_app5.pdf]

Multimedia Appendix 5. Health support systems – overview of GIS articles

Research Country and reference	Aims	Key effects
Vanuatu [77]	To examine a modern approach to GR using digital geospatial survey technology and explore its application in contemporary malaria elimination programmes.	Provided an effective and efficient operational tool for rapidly defining the spatial distribution of target populations in designated malaria elimination zones.
Australia [119]	To provide a commentary on the use of GIS as a tool for community coalitions and discuss some of the potential benefits and issues surrounding the development of these tools.	No findings
UK [121]	To describe a much better way of presenting Hansard table data in the form of interactive Web maps in Scalable Vector Graphics (SVG) format to further support health planners and decision makers in their planning and management tasks.	The interactive maps have the potential of further supporting health planners and decision makers in their planning and management tasks by allowing them to graphically interrogate data, instantly spot trends, and make quick and effective visual comparisons of geographically differentiated phenomena between different geographical areas and over time.
USA [122]	(1) To develop tools for health care professionals and communities to assess environmental exposures and (2) to evaluate the utility of integrating patient-reported environmental health information with GIS mapping of environmental data in a pilot study.	GIS was found to be a useful tool in displaying environmental risk factors and potentially associated health effects.

USA [123]	Evaluation of SOVAT: an OLAP-GIS decision support system for community health assessment data analysis.	Using SOVAT, tasks were completed more efficiently, with a higher rate of success, and with greater satisfaction, than the combined use of SPSS and GIS.
Canada [124]	To design an interoperable service oriented architecture based on Open Geospatial Consortium specifications to share the spatio-temporal disease information.	It enables cross-border visualization, analysis, and sharing of infectious disease information through interactive maps and/or animation in collaboration with multiple partners via a distributed network. It enables data sharing and users' collaboration in an open and interactive manner.
Canada [125]	To combine GIS with a web-based graphical user interface (webGUI) in a SDSS tool.	Health-care planners can model multiple scenarios to determine the optimal location for health services, as well as the number of people served in each instance.
France [126]	To describe the implementation of a Web-GIS to support public health decision making for ESRD.	No findings
Zambia [127]	To provide a review of data related to the operational use of a GIS-based DSS for optimal deployment, monitoring, and evaluation of entomological interventions for malaria control in Zambia.	The use of GIS has enabled detection of spatial trends of parasite prevalence following extensive deployment of front line vector control interventions; improved the tracking of entomological indicators: species characterization and insecticide resistance status, including parasite prevalence and impact assessment of ITNs and IRS.
Vanuatu [128]	To evaluate the user acceptability of a SDSS guiding IRS interventions.	Empowered programme managers at the provincial level to implement and asses the IRS intervention with the degree of detail required for malaria

		elimination.
Mexico [129]	To demonstrate that Google Earth™ can be used to strengthen overall public health capacity through development of information for city infrastructure and to display public health data in map formats.	It was demonstrated that a basic representation of city infrastructure useful as a spatial backbone in a DDSS can be rapidly developed at minimal cost.
Saudi Arabia [130]	To explore the possibilities of using GIS for private hospitals at Jeddah city, Saudi Arabia.	Helped health planners on evaluating the spatial distribution of hospital demand and for defining hospital service area.
