# Supplementary material for: Mobile Technologies and Geographic Information Systems to Improve Health Care Systems: A Literature Review
Source: JMIR Mhealth Uhealth. 2014 May 8;2(2):e21. doi: 10.2196/mhealth.3216 (PMC4114429; doi:10.2196/mhealth.3216)
Supplement: Supplementary file 6 [file mhealth_v2i2e21_app6.pdf]

Multimedia Appendix 6. Health promotion and disease prevention – overview of mHealth articles

Research Country and reference	Sample size	Device	Aims	Findings
Ireland [2]		SMS	To audit non-attendance rates at ENT out-patient clinics following the introduction of a text reminder system.	Sending text message reminders is a simple and cost-effective way to improve non-attendance at ENT outpatient clinics
USA [132]	70	SMS	To evaluate the effectiveness of cellular telephone text messaging as a reminder tool for improving adherence to sunscreen application.	SMS technology offers an innovative, low- cost, and effective method of improving adherence to sunscreen application.
USA [133]	195	SMS	To determine the impact of text message immunization reminder-recalls in an urban, low-income population.	The intervention improved immunization coverage in a low-income, urban population.
USA [134]	10	Mobile phone	To evaluate user satisfaction with the integrated system, including the potential of the device to transmit self-monitoring data to a website for review and analysis by clinicians, parents, and patients	Adolescents liked the integration of the two technologies and agreed that the glucometer was easy to use and that the tool was useful in the management of their diabetes.
USA [135]	50	SMS	To test the feasibility of using automated SMS technology to monitor appetite ratings hourly over seven consecutive days.	Automated text-messaging is a feasible method to monitor appetite ratings.
USA [136]	20	SMS	To examine the use of tailored messages sent to	Computerized tailored SMS is a feasible adjunct to

			obese adolescents enrolled in a weight management programme, as a means of increasing adherence.	multidisciplinary obesity treatment. Adolescents perceived the intervention as an enjoyable means of improving their adherence to healthy lifestyle practices.
USA [137]	95	Mobile phone: voice	To assess the efficacy of an innovative smoking cessation (SC) intervention targeted to a multiethnic, economically disadvantaged HIV-positive population.	Patients were receptive to, and could be helped by SC treatment. Counseling delivered by cellular telephone, can significantly increase smoking abstinence rates over that achieved by usual care.
USA [138]	95	Mobile phone	To assess the effects of a cell phone intervention (CPI) on hypothesized mediators demonstrated to influence cessation outcomes.	CPI group experienced greater reductions in anxiety and depression, and increases in self-efficacy.
USA [139]	95	SMS	To investigate the feasibility of SMS to enable ongoing communication with African-American women participating in a weight management program.	Early feasibility and acceptability of SMS as a method for promoting healthy behaviors for weight maintenance.
USA [140]	9213	SMS	To evaluate targeted SMS reminders for low-income, urban parents to promote receipt of influenza vaccination among children and adolescents.	The SMS intervention compared with usual care was associated with an increased rate of influenza vaccination. However, the overall influenza vaccinatio n rate remained low.
USA [141]	1200	SMS	To evaluate the effect of SMS on attendance following	Text messaging resulted in a modest improvement

			male circumcision for HIV prevention.	in attendance at the 7-day post-operative clinic visit following adult male circumcision.
UK [142]		SMS	To assess the effectiveness of a text message result service within an inner London sexual health clinic.	The intervention resulted in saving of staff time per month; reduced number of days to diagnosis.
UK [143]	288	Mobile phone	To determine whether mobile phone based monitoring improves asthma control compared with standard paper based monitoring strategies.	Mobile technology does not improve asthma control or increase self-efficacy compared with paper based monitoring. The mobile technology was not cost-effective.
UK [144]		Mobile phone	To describe the development and implementation of an innovative real-time telemedicine system based around transmission and feedback of data to and from a mobile phone.	Patients had immediate feedback. Clinicians supporting patients had access to summary screens identifying users not testing, and those with levels of blood glucose outside pre-defined limits.
UK [145]	11	Mobile phone: GPS	To describe the use of global positioning system (GPS) technology to locate missing persons with dementia.	When compliance was high, the tracking system was highly reliable and accurate.
UK [146]	21	SMS	To explore the feasibility, acceptability, and efficacy of a SMS-based intervention in the aftercare of bulimia nervosa patients who had received outpatient psychotherapy.	Levels of use of the program were relatively low and attrition high, indicating limited acceptance of the intervention.
UK [147]	201	SMS	To determine the	Routine SMS texting is a

			effectiveness of a SMS reminder in improving attendance in return general ophthalmology clinic patients	cost-effective means of reducing DNAs and should become standard practice.
UK [148]	173	SMS	To evaluate the effectiveness of texting appointment reminders to patients who persistently fail to attend appointments.	No significant reduction in non-attendance rates, as a result of texting appointment reminders to patients who persistently fail to attend their general practice appointments.
UK [149]	276	SMS	To evaluate the operational and financial efficacy of sending SMS to patients with outpatient clinic appointments.	The use of SMS as a reminder may reduce the failure to attend rate for outpatient paediatric dental appointments.
UK [150]	447	SMS	To assess the effectiveness of the use of SMS-based reminders for hospital outpatient appointments as a method of reducing the non-attendance rates in an inner-city London teaching hospital	The intervention was associated with a reduction of 38% in the likelihood of patients not attending their appointments, compared to no appointment reminder. The SMS may also be more cost-effective than traditional appointment reminders and require less labour.
UK [151]		SMS	To (1) assess whether SMS reminders would reduce non-attendance at a paediatric outpatient clinic; (2) clarify the families' attitude towards receiving an SMS reminder.	SMS reminders reduced non-attendance at the paediatric outpatient clinic.
UK [152]	276	Mobile phone	To assess the patterns and dynamics of mobile phone	Mobile phones have a potential for use in

			usage amongst an ART cohort in rural Uganda a nd ascertain its feasibility for improving clinic attendance.	resource-constrained settings to substantially improve the clinical management of HIV/AIDS.
UK [153]			To assess whether the use of SMS reminders sent to patients prior to their dental appointments improved attendance rates for two dentists at a dental access centre.	SMS appointment reminders reduced the number of failed appointments significantly. They can provide an automated, non-intrusive, and cost-effective method of improving patient attendance at dental appointments.
UK [154]	2817	SMS	To examine the effect of SMS reminders on the attendance of appointments at four community mental health clinics.	SMS-based technology can offer a time-, labor-, and cost-efficient strategy for encouraging engagement with psychiatric outpatient services
Norway [155]	15	SMS	To sought an understanding of the tested system's potential role, feasibility of use in daily life, desired functionality, and approaches that may be relevant for future developments and research.	The system was easily integrated into everyday life and was particularly effective when children measured their blood glucose levels regularly.
Ireland [156]	6	SMS	To evaluate the accuracy and viability of a mobility telemonitoring system, based on the SMS, to monitor the functional mobility of elderly subjects in an unsupervised environment.	Strong positive correlation's were found for the detection of sitting (r = 0.996) and for the combined postures of standing and walking (r = 0.994). Subjects suggested that a

				lighter, smaller and wireless unit would be more effective.
Ireland [157]		SMS	To describe a SMS-based mobility monitoring system that can monitor a subject independently of his or her location.	It provides long-term data on which clinicians can base their clinical decisions. The system also has the potential to reduce the financial burden on health care services by facilitating more efficient use of health care resources.
Netherlands [158]	141	SMS	To analyze whether self-monitoring of lifestyle behaviours through a SMS maintenance treatment (SMSMT) with personalized feedback positively effects weight, lifestyle behaviours and psychological well-being in obese children.	There was no positive effect of SMSMT on weight, eating behaviour or psychological well-being in obese children. SMSMT seems to be a feasible method of treatment delivery.
Netherlands [159]	141	SMS	To explore the effect of an SMS approach aimed at improving treatment results and reducing dropout rates in a pediatric lifestyle intervention.	SMS maintenance treatment is effective in reducing dropout rates from a pediatric lifestyle intervention.
Austria [160]		Mobile phone	To provide an easy way for documenting the course of psoriasis during acute episodes as well as during remission phases by the use of an electronic therapy diary.	Mobile phone-based acquisition of images of dermatological lesions by patients themselves is feasible with sufficient quality for therapy assessment.
New Zealand	1705	SMS	To determine whether a	A mobile phone-based

[161]			smoking cessation service using mobile phone text messaging is as effective for Maori as non-Maori.	cessation programme was as effective for Maori as non-Maori at increasing short-term self-reported quit rates.
New Zealand [162]	1705	SMS	To determine the effectiveness of a mobile phone SMS smoking cessation programme.	A SMS based smoking cessation programme doubled quit rates at six weeks.
Australia [163]	1771	SMS	To determine the impact of using SMS on a population level on sexual health knowledge and behavior.	SMS appear to be a feasible, popular, and effective method of sexual health promotion to young people with a relatively low withdrawal rate, positive feedback, and an observed improvement in sexual health knowledge and STI testing.
Australia [164]	2151	SMS	To evaluate the effect of appointment reminders sent as SMS text messages to patients' mobile telephones on attendance at outpatient cli nics.	SMS may be a suitable means of improving patient attendance.
Australia [165]	22658	SMS	To evaluate the operational and financial efficacy of sending short message service (SMS) text message reminders to the mobile telephones of patients with scheduled outpatient clinic appointments.	The FTA reduction coupled with the increase in patient revenue suggests that reminding patients using SMS is a very cost effective approach for improving patient attendance.
Kenya [166]	119	SMS	To assess whether SMS reminders sent to health	In resource-limited settings, malaria control

			workers' mobile phones could improve and maintain their adherence to treatment guidelines for outpatient paediatric malaria.	programmes should consider use of text messaging to improve health workers' case-management practices.
Croatia [167]	16	SMS	To examine Global System for Mobile Communications mobile telephone SMS as a novel means of telemedicine in Home peak expiratory flow monitoring.	SMS is a convenient, reliable, affordable, and secure means of telemedicine that may improve asthma control whe n added to a written action plan and standard follow-up.
Singapore [168]	120	SMS	To evaluate the feasibility of using the mobile phone SMS for symptom monitoring in patients with asthma.	There was no reduction in admission rates in either group. The service was accepted by most patients.
Brazil [169]		SMS	To present the results of a study on the impact of appointment reminders sent as SMS to patients' cell phones on nonattendance rates at outpatient clinics.	Sending appointment reminders as SMS to patients' cell phones is an effective strategy to reduce nonattendance rates.
China [171]		SMS	To design a product and service for Chinese BP measurement needs with special focus on Community Healthcare Centers/Points.	No findings
China [172]	1859	SMS, voice	To compare the efficacy of a SMS text messaging and phone reminder to improve attendance rates at a health promotion center.	SMS and call are effective reminders for improving attendance rate at a health promotion center. SMS reminder may be more cost-effective compared with the call reminder.

Korea [173]	51	SMS	To investigate the effectiveness of an educational intervention that used both the cellular phone and the Internet to provide a SMS relating to plasma glucose levels.	This educational intervention using the Internet and an SMS by cellular phone improved levels of HbA(1)c and 2HPMG.
Korea [174]	51	SMS	To investigate the effectiveness of a nurse SMS by cellular phone and wire Internet on plasma glucose levels in people with diabetes for six months.	This web-based intervention using SMS of cellular phone improved HbA1c and 2HPMG for six months in type-2 diabetic patients.
India [176]	106	SMS	To find out the effectiveness of SMS as a reminder system for regular practice of BSE by women.	SMS increased the practice of breast self-examination.
Malaysia [178]	993	SMS	To determine the effectiveness of a text messaging reminder in improving attendance in primary care.	Text messaging reminder system was effective in improving attendance rate in primary care. It was more cost-effective compared with the mobile phone reminder.
Malaysia [179]	931	SMS	To determine if SMS would be effective in reducing non-attendance in patients on long-term follow-up, compared with telephone reminders and no reminder.	Text messaging was found to be as effective as telephone reminder in reducing non-attendance in patients who required long-term follow-up for their chronic illnesses.
Australia [278]	43	SMS	To present the results of evaluation focus groups among participants who received twelve sexual health	Text messages were viewed as an acceptable and ‘personal’ means of health promotion, with participants

				related SMS as part of a study examining the impact of text messaging for sexual health promotion to on young people in Victoria, Australia.	particularly valuing the informal language. There was a preference for messages that were positive, relevant and short and for messages to cover a variety of topics.
