# Supplementary material for: Mobile Technologies and Geographic Information Systems to Improve Health Care Systems: A Literature Review
Source: JMIR Mhealth Uhealth. 2014 May 8;2(2):e21. doi: 10.2196/mhealth.3216 (PMC4114429; doi:10.2196/mhealth.3216)
Supplement: Supplementary file 7 [file mhealth_v2i2e21_app7.pdf]

Multimedia Appendix 7. Health promotion and disease prevention – overview of GIS articles

Research Country and reference	Aims	Key effects
USA [131]	To identify community settings most likely to reach individuals from geographically localized areas.	Some community settings are more likely than others to reach highly localized populations.
Italy [181]	To identify regional spots as potential territorial stations for the telemedicine service.	No findings
Canada [182]	1) to assess the prevalence and incidence of psychological distress, mental disorders, substance abuse, parasuicide, risky behaviour and quality of life; 2) to examine the links and interactions between individual determinants, neighbourhood ecology and mental health in each neighbourhood; 3) to identify the conditions facilitating the integration of individuals with mental health problems; 4) to analyse the impact of the social, economic and physical aspects of the neighbourhoods using a geographic information system. 5) to verify the adequacy of mental health services.	Several vulnerable groups and risk factors related to socio-demographic variables have been identified such as: gender, age, marital status, income, immigration and language.
USA [183]	To use GIS to create a site selection strategy for the dissemination and pilot evaluation of a community-based fall prevention program for older adults.	GIS can be used to determine actual travel time, and may facilitate the selection of community-based prevention program sites to maximize accessibility and utilization by targeted populations.

Brazil [184]	To analyze dental trauma using a geographic information system as a tool for integrating social, environ- mental and epidemiological data.	The spatial analysis confirms the hypothesis that there is significant variation in the occurrence of dental trauma, considering the place of residence in the population studied.
China [185]	To study spatial distribution rules and risk factors for syphilis.	Temperature, distance from railways and highways, and the normalised difference vegetation index were deter- mined as supporting variables with regard to the transmission of the disease by both univariate and multivariate spatial correlation analyses.
Ireland [186]	To utilise a GIS for plotting measles cases on a digital map in real time.	The digital mapping documented the evolution of two distinct clusters of 87 (35%) cases. The two clusters occurred in socio-economically disadvantaged areas and were attributable to inadequate measles vaccination coverage due in part to the interruption of a school-based MMR2 vaccination programme.
USA [187]	To use GIS for visualization of cancer risk patterns associated with incidence, mortality, and accessibility to care.	The mapping of cancer incidence, mortality, and staging, transportation access, and multiple layers of content were found to have significant associations with perceived research advantages.
USA [188]	To describe our initial experience using an animated geographic information system (GIS) to inves- tigate factors associated with nosocomial transmission of resistant organisms.	Animated GIS can uncover previously hidden factors that contribute to the spread of nosocomial infections. This tech- nology may become a useful adjunct for the prevention of nosocomial transmission of infectious agents.

USA [189]	To locate all out-of-hospital cardiac arrests (OHCAs) and identify clusters of OHCAs, as well as clusters of patients who did not receive bystander cardiopulmonary resuscitation (CPR), in order to identify locations that may benefit from prevention efforts.	Clusters of OHCAs can be identified, which could be used to guide resource allocation. Clusters of OHCAs in which the patients did not receive bystander CPR can also be identified and could be used to direct educational programs.
USA [192]	To identify geographic areas with elevated risk for the later development of ALS among military personnel who served in the first Gulf War.	Specific geographic locations of troop units within the 1991 Gulf War theatre are associated with an increased risk for the subsequent development of ALS among members of those units.
Papua New Guinea [193]	To use GIS technology to map the prevalence of malaria in the Wosera Health and Demographic Surveillance Site.	Malaria is endemic with high prevalence as observed across the 3 surveyed years.
India [194]	To identify the malaria hot spots at health subcentre level in an endemic district using a GIS.	GIS identified 10 hot spots at extremely high risk of malaria and 14 hot spots at high risk of malaria. The GIS model used in this study can be used, even at village or cluster level, to pin point the malaria hot spots, and information can
Kenya [195]	To describe an electronic injury surveillance system that provides data for improving patient care and monitoring injury incidence and distribution patterns	Digital maps of injury spatial distribution were created using GIS software and correlated injury type and location with patients' clinical data.
Canada [196]	To examine sex-specific spatial patterns of overweight/ obesity in Canada as well as investigates the presence of spatial clusters.	Results revealed marked geographical variation in overweight/obesity prevalence with higher values in the Northern and Atlantic health-regions and lower values in the Southern and

		Western health- regions of Canada. Significant positive spatial autocorrelation was found for both males and females, with significant clusters of high values or 'hot spots' of obesity in the Atlantic and Northern health-regions of Alberta, Saskatchewan, Manitoba and Ontario.
Japan [197]	To explore the impact of the intervention coverage and people's adherence to the intervention on malaria health outcome among targeted villages in various geographic locations.	Based on the data and maps, it was demonstrated that malaria remained unevenly distributed within districts.
USA [198]	To quantify the relationship between gonorrheal infection rates in California and a measure of poverty status and investigated how this relationship and the spatial dispersion of cases varied among the 4 dominant racial/ethnic groups in the state.	There was a strong positive relationship between poverty and infection, but racial/ethnic disparities in infection, driven by a disproportionate level of gonorrhea among African Americans, eclipsed this differential. The degree of spatial aggregation varied substantially among groups and was especially pronounced for African Americans with gonorrhea in the highest poverty category.
India [199]	To present a spatial mapping and analysis of filariasis data over a 3-year period (2004–2007) from Karimnagar, Chittoor, East andWest Godavari districts of Andhra Pradesh, India.	It was demonstrated that filariasis remained unevenly dis- tributed within the districts.
China [200]	To combine ecologic niche modeling with GIS and remote sensing techniques to identify the risk factors	Land cover and elevation were found to be closely associated with the presence of hantavirus-infected

	and affected areas of hantavirus infections in rodent hosts.	rodent hosts. The aver-aged area under the receiver operating characteristic curve was 0.864, implying good performance.
