# Supplementary material for: Mobile Technologies and Geographic Information Systems to Improve Health Care Systems: A Literature Review
Source: JMIR Mhealth Uhealth. 2014 May 8;2(2):e21. doi: 10.2196/mhealth.3216 (PMC4114429; doi:10.2196/mhealth.3216)
Supplement: Supplementary file 8 [file mhealth_v2i2e21_app8.pdf]

Multimedia Appendix 8. Communication to or between health care providers – overview of mHealth articles

Research Country and reference	Sample size	Device	Aims	Findings
Kenya [57]		PDA	To assess the characteristics and outcomes of patients with acute respiratory illnesses (ARIs) visiting a rural health center, using an existing electronic medical record (EMR) system and personal data assistants (PDAs).	EMRs and PDA are useful tools for performing prospective clinical research in resource constrained developing countries.
USA [90]	40	SMS; email	This pilot and feasibility study compared two-way SMS cell phone messaging wi th e-mail reminders that were directed at encouraging blood glucose (BG) monitoring.	Cell phone text messaging to promote BG monitoring is a viable and acceptable option in adolescents and young adults with diabetes.
Italy [205]	18	Mail, SMS	To describe a store-and-forward teleconsultation network developed as a web application in order to provide second opinions on retinal diseases	The system was rated as very good or good for Access, Acceptability, Image Quality and Medical Efficacy.
USA [206]	10	Mobile phone	To examine the feasibility and acceptability of cell phone use in individuals with schizophrenia spectrum disorders (SSDs)	Majority of individuals with SSDs are willing to use this method of communication and are able to do so with few problems
USA [207]	83	Mobile phone	To determine the extent and scope of cell phone use by 83 adults with intellectual	There is a gap in the use of cell phone technology between people

			disabilities, reasons for nonuse, and factors affecting use.	with intellectual disabilities. When used, they are employed primarily for emergencies, storing telephone numbers, and day-to-day communication.
USA [208]		PDA	To assess how PDA augmented medical care during street outreach to the homeless in a major metropolitan area.	PDA free clinicians to focus on building relationships instead of recreating documentation during patient encounters
USA [209]	366	PDA	To identify trends in the utilization and acceptance of handheld computers (personal digital assistants) among medical students during preclinical and clinical training.	The students considered handheld computing an essential technology that was integral to their performance as future clinicians.
USA [210]	105	PDA	To assess both confort levels and self-described skill level before and after medical students class.	PDA class is beneficial, necessary, and should remain mandatory to faculty, students, and instructors.
USA [211]	60	PDA	To investigate the current personal digital assistant (PDA) usage patterns of the residents and their interest in future PDA-based applications	Our data show current trends of use of PDAs by Pediatrics and emergency Medicine residents and their interest for future applications.
USA [212]		PDA	To assess clinical reasoning when PDAs are used as an information resource for nursing students.	PDAs improve nursing work force, including accurate differential diagnosis and diagnostic reasoning,

				reduction of medication errors, reduction of healthcare costs, and development of effective treatment protocols.
USA [213]	11	SMS	To examine African American adolescents' perceptions of a mobile cell phone (MCP)-enhanced intervention and development of an MCP-based HIV prevention intervention.	Adolescents said they benefited from the MCP-enhanced approach and were receptive to the idea of developing an MCP-based intervention.
USA [214]	47	SMS	To assess the feasibility of engaging adults with diabetes in self management behaviors between clinic visits by using cell phone text messaging to provide blood sugar measurement prompts and appointment reminders.	Cell phone-based text messaging may enhance chronic disease management support and patient-provider communications beyond the clinic setting
USA [215]	389	PDA	To determine the benefit, if any, from use of and access to PDAs on the medical epidemiology final examination.	Use of PDAs and whether students thought they might be helpful had no measurable effect on performance on an epidemiology final examination.
USA [216]	223	Mobile phone	To develop and test a theoretical model that explains the underlying process through which the use of cell phones can facilitate the capacity of community health care	Access to institutional resources was positively associated with midwives' health knowledg e. Further, access to peer resources was associated with higher self-efficacy,

			workers in developing regions	which was positively associated with health knowledge.
USA [217]	209	Mobile phone	To evaluate the feasibility of using information communication technology-based interventions to improve access to HIV testing and education within the Spanish-speaking community in Baltimore.	Interest was high for receiving health education, HIV edu cation, and test results via text messaging.
USA [218]	12	PDA	To determine patients' perceptions of physicians' personal digital assistant (PDA) use, comparing the results across 8 physician-patient dimensions important to clinical interactions.	Patients rate interactions with their physicians more positively when physicians explain their PDA use.
USA [219]	4203	PDA	To comprehensively characterize the current use of electronic health records (EH Rs) and PDAs among family physicians in Florida; and to compare family physicians with other doctors with respect to the functions commonly used on their EHR and PDA systems.	Even though no differences in overall HER adoption were found, family physicians, when compared with other physicians, were significantly more likely to be using a more robust set of EHR functions which include allergy and medication lists, diagnosis, problem lists, patient scheduling and educational materials, preventive services reminders and access to reference

				material.
USA [220]	17	Mobile phone	To determine the feasibility and efficacy of a six-month, cell phone-based exercise persistence intervention for patients with chronic obstructive pulmonary disease (COPD) following pulmonary rehabilitation.	It is feasible to deliver a cell phone-based exercise persistence intervention to patients with COPD post-rehabilitation and that the addition of coaching appeared to be no better than self-monitoring.
USA [221]	315	SMS	To assess knowledge, attitudes, and behaviors surrounding healthcare-related mobile phone use and text messaging among persons at risk for or infected with tuberculosis (TB) or the human immunodeficiency vir us (HIV).	Cell phones and text messa ging were prevalent among our subjects attending HIV and TB clinics, and subjects were generally receptive to text messaging for healthcare-related communication.
USA [222]	10	PDA	To describe the use of (1) PDAs in patient care and (2) a PDA learning portfolio intended to encourage documentation of reflection on practice and medical education.	PDA can facilitate accessing drug information and clinical guidelines and using this information to make clinical decisions.
USA [223]	169	Mobile phone	To conduct a preliminary examination of cell phone ownership and use among a sample of homeless youth, particularly the social and instrumental uses of cell phones which have implications for health outcomes.	Cell phones present new opportunities for intervention research, connecting homeless youth to family and home-based peers who can be sources of social support in times of need.

USA [224]	1839	SMS	To examine both the correlates of sexting and the association of sexting with physical sexual risk behavior among a representative probability sample of high school students.	Sexting, rather than functioning as an alternative to “real world” sexual risk behavior, appears to be part of a cluster of risky sexual behaviors among adolescents.
USA [225]	439	Mobile phone	To (1) determine the frequency of cell phone use in the perfusion community, and (2) to identify concerns and opinions among perfusionists regarding cell phone use.	Majority of perfusionists believe cell phones raise significant safety issues while operating the heart-lung machine. However, the majority also have used a cell phone while performing this activity. There are clear generational differences in opinions on the role and/or appropriateness of cell phones during bypass.
USA [227]		PDA	To discuss the utilization of PDAs for Family Nurse Practitioner students in a Pediatric course	The PDA made it possible for students to utilize pre-existing knowledge with additional learning resources
USA [228]	648	PDA	To assess the use of personal digital assistants (PDAs) among dietitians and dietetic students and the desire for training in the use of PDAs.	Dietitians and students expressed an interest in receiving training in PDA use, preferably from state and local dietetic associations.
UK [229]	10	Mobile phone	To study of the attitudes of patients and staff to the use of mobile phone technology for	Mobile phone was seen as identifying poor control more quickly and

			recording and gathering asthma data	facilitating communication with healthcare professionals without the need for face-to-face consultation. There was a high degree of acceptability to both patients and staff.
UK [230]		Mobile phone	To present details regarding the early developments of a memory prompt solution for persons with early dement ia.	No findings
UK [231]	14	Mobile phone: camera	To determine the accuracy of assessment of common ENT emergency radi ological investigations using mobile phone digital images.	The accuracy and confidence of diagnosis of common ENT emergency r adiological investigations using mobile phone digital images seem compatible with X-ray box. It is cost-effective. It may be used to augment information sent to non-resident specialists in the management of emergency ENT cases.
UK [232]		SMS: Sweet Talk	To explore how patients with type 1 diabetes interact with the Sweet Talk system in order to understand its utility to this user group.	Automated, scheduled text messaging successfully engaged young people with diabetes.
France [233]	10	Mobile phone: MMS	To investigate the application and assessment of the usefulness of image transfer through a mobile telephone in pediatric orthopaedic	Teleconsultation using MMS is especially useful to improve remote management of orthopaedic patients in

			practice.	local hospitals or for decisions of transfer when surgical treatment is needed.
Canada [234]		PDA	To outline the development and evaluation of a wireless PDA based clinical learning tool designed to promote professional reflection for health professionals.	Nurse practitioner and medical students revealed positive attitudes to the use of PDA based tools and portfolios, but limits to the use of the PDA portfolio due to the inherent interface restrictions of the PDA.
Canada [235]		PDA	To explore advanced practice nurses' perceptions on wireless PDA technologies, to establish the type and range of tools that would be useful to support their practice and to identify any requirements and limitations that may impact the implementation of wireless PDA in practice.	Nurses identified improved client care as the major benefit of this technology in practice and the type and range of tools they identified included clinical reference tools such as drug and diagnostic/laboratory reference applications and wireless communicatio ns.
Canada [236]	9	PDA	To develop and evaluate a decision support tool (DST) on a PDA for cardiac tele-triage/tele-consultation when the presenting problem was chest pain.	The DST is useful to advanced cardiac care nurs es; it has potentiality for use by nurses less experienced in cardiac care, and it has potentiality for use in an interdisciplinary team environment.
Canada [237]	23	PDA	To	Physical therapists prefer

			explore physical therapists' preferences for strategies to facilitate their access to, evaluation and implementation of the stroke research literature in clinical practice.	technology-assisted access to resources and educationand favour attending multiple interactive, expert-facilitated education sessions incorporating opportunities for case-based learning and practice of new skills to change behaviour related toevidence-based practice.
Japan [238]	215	Mobile phone: email	To classify the trends of health information of pregnancy and delivery in which people desire to obtain through cell phone based on the characteristics of the information and, of each classification we revealed the generic characteristic (age, gender, gestational weeks and any other children), any social support, the types of cell phones or/and computers and their related use.	Providing medical information and non-medical information through cell phone e-mail is an essential service, especially in Japan.
Australia [239]	647	PDA	To present a snapshot of student opinion on the usefulness of the RMRPDA service as an adjunct teaching and learning resource during their RMR clinical	Students highly agreed that access to PDA information resources contributed to learning while on rural placements.

			placements.	
New Zealand [240]	596	SMS	To assess the impact of text messaging as the preferred method of communicating positive Chlamydia trachomatis test re sults in an urban sexual health clinic.	The use of this technology is as effective as more traditional means of communication. The increase in cases of C trachomatis treated within 4 weeks may reflect the significant increase in mobile phone use and improved ability to contact people rather than simply the introduction of text messaging.
Denmark [241]	2550	Mobile phone	To examine the association between a mobile phone intervention and skilled delivery attendance in a resource-limited setting.	The mobile phone intervention significantly increased skilled delivery a ttendance amongst women of urban residence.
Thailand [242]	59	Mobile phone: MMS	To investigate the accuracy and usefulness of teleconsultation using the mobile phone MMS in emergency orthopaedic patients.	Teleconsultation via MMS demonstrated good reliability, but poor diagnostic accuracy which could have major consequences in emergency orthopaedic patients.
Peru [243]	15	Colect a-PALM : PDA	To test the usability of a Web-based application delivered on PDAs that provides behavioral messaging based on risk assessment responses for HIVpatients.	Users found the tool innovative, interesting, easy to use, educational, trustworthy, private, and non-judgmental. Colecta-PALM was well-received and easily usable by most.

Kenya [244]	538	SMS	To assess whether mobile phone communication between health-care workers and patients starting antiretroviral therapy in Kenya improved drug adherence and suppression of plasma HIV-1 RNA load.	Patients who received SMS support had significantly improved ART adherence and rates of viral suppression compared with the control individuals. Mobile phones might be effective tools to improve patient outcome in resource-limited settings.
Kenya [245]	538	Mobile phone	1) Describe problems participants identified through mobile phone support and reasons why participants did not respond to the messages; 2) investigate factors associated with indicating a problem and not responding; and 3) examine participant perceptions of the intervention.	The WelTel intervention enabled frequent communication between clinicians and patients during the WelTel Kenya1 trial. Many patients valued the service for the support it provided, with health-related concerns comprising the majority of problems identified by participants. Few
Taiwan [246]	35	Mobile phone: camera	To investigate the feasibility ofteleconsultation with the mobile camera-phone to transfer clinical images and communicate on line for evaluation of replantation potential in completely amputated fingers.	The camera-phone is a feasible tool for remote evaluation regar ding the replantation potential of completely amputated fingers and it holds significant promise in avoiding unnecessary patient transfer by providing useful information.

Taiwan [247]		Mobile phone	To demonstrate transmission of 12-lead electrocardiography (ECG) in an ambulance to the cell phone of the attendant emergency medical technician and then to the hospital and to cell phones of off-site cardiologists	The 12-lead ECG transmission e-technology expands the functions of a 12-lead ECG instrument and facilitates more efficient pre-hospital cardiac care.
Korea [248]	927	SMS	To conducte a community-based anti-obesity programme using mobile pho ne SMS messaging.	Majority of participants were satisfied with the weekly SMS messages and information brochures delivered by post. SMS messaging may be an effective method of behaviour modification in weight control and anti-obesity health education programmes when promoted by community health centres.
Korea [249]		PDA	To examine an instant image transfer system based on a PDA phone with a built-in camera. Images displayed on a picture archiving and communication systems (PACS) monitor can be captured by the camera in the PDA phone directly.	Although higher resolution produced higher clinical satisfaction, it also required more transmission time. At the limited bandwidth employed, higher resolutions could not be justified.
Korea [250]	138	Mobile phone: camera	To assess the feasibility of using a camcorder mobile phone for	Immediate basic teleconsul ting of echocardiography movies is possible using

			teleconsulting about cardiac echocardiography.	current commercially-available mobile phone sys tems
Korea [251]		Mobile phone: camera	To evaluate the success rate and time to transfer images under various conditions.	The image quality of most built-in cameras was acceptable to perform the initial assessment, with the autofocus function being essential to obtain high-quality images. The transmission failure rate increased markedly when the image size exceeded 500 KB and the additional text messaging did not improve the success rate or the transmission time.
Korea [252]	51	Mobile phone	Investigate the effectiveness of an educational intervention that used both the cellular phone with a short messaging service (SMS) and the Internet on the glycemic control of the patients with type 2 diabetes mellitus.	This educational intervention using the Internet and a SMS by cellular phone rapidly improved and stably maintained the glycemic control of the patients with type 2 diabetes mellitus.
Israel [253]	45	PDA	To determine residents' perception of the utility of personal digital assistants (PDAs) and their influence on clinical practice at two teaching hospitals, one of which subsidized resident purchase of a PDA.	Palm owners in both groups responded that they used these devices to organize their record keeping and the most frequently used programs were pharmacopoeias, medical reference and clinical calculators.
Israel [254]	120	Mobile	To assess the attitudes of	They preferred to answer

		phone; email	physicians to providing their telephone number or email address to patients.	their cell phones only during the daytime and at predetermined times, but would answer email most hours of the day, including weekends and holidays (P = 0.001). More physicians (79.7%) would have preferred allotted time for email communication than allotted time for cell phone communicat ion (50%). However, they felt that email communication was more likely to lead to miscommunication than telephone calls (P = 0.0001).
Israel [255]	200	Moile phone; email	To assess the attitudes and practice of patients on obtaining thecell phone number or e-mail address of their physician for the purpose of medical consultation.	Patients favored cell phone s over e-mail for consulting with their treating physicians.
Uganda [256]	1503	Mobile phone	To better understand the potential to deliver cell phone-based HIV prevention programs to adolescents in re-source limited settings	61% of those who owned a cell phone believe that they would access a text messaging-based HIV prevention program if it were available. Other forms of program delivery modality (e.g. Internet,

				religious organizations, schools) were preferred to text messaging however.
Uganda [257]	50	SMS	To determine: a) cell phone use practices and literacy, b) preferences for laboratory result communication, c) privacy and confidentiality, and d) acceptability of and preferences for text messaging of health information.	Cell phone text messaging for communication of abnormal laboratory results is highly acceptable in this cohort of HIV-infected patients in rural Uganda
Thailand [258]		PDA	To assess the accuracy of diagnosis, cost and the transmission time to a PDA phone.	Good accuracy can be achieved with a low-cost system for teleradiology consultation in stroke.
Singapore [259]		SMS	To report and discuss the critical values of a novel, fully automated short message system (critical reportable result health care messaging system [CRR-HMS]), and the performance of the automated reporting system.	The CRR-HMS is a clinically useful tool to rapidly communicate critical results to targeted physicians to facilitate rapid and timely intervention.
Turkey [260]		PDA	To evaluate 11 commercial laboratory medicine software products.	The use of PDAs will facilitate medical applications, constitute an information base for new learning and make learning more pleasant.
India [261]	173	SMS	To compare the effectiveness of two media (text messages and pamphlets) in imparting health education to	Text messaging was more effective than pamphlets in improving knowledge, attitude and practices

			mothers of preschool children.	of mothers, but the comparative reduction in plaque score between groups was not significant. Text messagin g appears to be an effective means of imparting oral health educa tion.
South Africa [262]		Mobile phone	To obtain information on the: (1) subjective responses of therapeutic counsellors to the technology; (2) communication between the counsellors and health services; (3) patient's responses	Cell-Life has improved the efficiency of communication between patient, counsellor and healthcare worker.
