# Supplementary material for: Mobile Technologies and Geographic Information Systems to Improve Health Care Systems: A Literature Review
Source: JMIR Mhealth Uhealth. 2014 May 8;2(2):e21. doi: 10.2196/mhealth.3216 (PMC4114429; doi:10.2196/mhealth.3216)
Supplement: Supplementary file 9 [file mhealth_v2i2e21_app9.pdf]

Multimedia Appendix 9. Communication to or between health care providers – overview of GIS articles

Research Country and reference	Aims	Findings
USA [263]	To provide a synopsis of some suggestions and comments by practitioners on how best to communicate the results of spatial analyses of health data.	The biggest challenge is to ensure that maps of health statistics inform without misinforming. Advances in the sciences of cartography, statistics, and visualization of spatial data are constantly expanding the toolkit available to mapmakers to meet this challenge. Asking potential users to answer questions or to talk about what they see is still the best way to evaluate the effectiveness of a specific map design.
USA [264]	The objective was to determine whether utilizing GIS could help a community to identify risk and develop potential interventions to address perinatal health problems.	This collaboration resulted in the development of a perinatal GIS model that helped community members to decide where to focus interventions and in continued use of GIS for planning.
USA [265]	To summarize a methodology and demonstrate the potential of this free and open standard to contribute to the dissemination of Expanded Program on Immunization (EPI) information by providing interactive maps to a wider audience through the Internet.	No findings
USA [266]	To describe the delivery of training work- shops to enhance the competencies of health workers in	Participants enhanced competencies and skills in biodefense informatics and data management. Self-reported

	biodefense informatics and discusses its implication for delivering education to rural regions.	evaluation indicated that knowledge increased upon completion of the training. The majority (97%) of the participants found the workshops relevant and useful, and many noted that the courses enhance their preparedness efforts.
USA [267]	To gain CCC program managers's insights about the compatibility of GIS mapping for CCC, the target audiences to be reached with maps as a CCC message, and relative advantages of this technology in its diffusion.	GIS use for cancer control has the potential to build community capacity and social capital for communities as a way to reduce the cancer burden.
UK [268]	(i) to explore how public health professionals perceive and value GIS technologies; (ii) to examine the advantages and disadvantages associated with the use of GIS outputs in public health decision-making; and (iii) to consider the potential for GIS as vehicles for data sharing and facilitating intra- and inter-agency collaboration.	Interviewees were positive about the value of GIS in public health practice but many were quick to express that they were not a panacea or wonder tool that could be used to inform decision-making in relation to all types of public health problems. Most interviewees were aware of both the advantages and disadvantages of using GIS outputs in decision-making.
UK [269]	To conduct a pilot study into the comprehension and visualisation preferences of geographic information by public health practitioners (PHPs), particularly in the context of interactive, Internet-based atlases.	Novice users of disease maps had difficulties in interpreting data classifications, in understanding supplementary information in the form of box plots and histograms, and in making use of links between interactive tabular and cartographic information. Choices for colour plans when viewing maps showed little agreement between

		users, although pre-viewing comments showed preferences for red-blue diverging schema.
