# Supplementary material for: Mobile Technologies and Geographic Information Systems to Improve Health Care Systems: A Literature Review
Source: JMIR Mhealth Uhealth. 2014 May 8;2(2):e21. doi: 10.2196/mhealth.3216 (PMC4114429; doi:10.2196/mhealth.3216)
Supplement: Supplementary file 10 [file mhealth_v2i2e21_app10.pdf]

Multimedia Appendix 10. Medical education – overview of articles mHealth articles

Country and reference	Sample size	Device	Aims	Findings
USA [209]	366	PDA	To identify trends in the utilization and acceptance of handheld computers (personal digital assistants) among medical students during preclinical and clinical training.	The students considered handheld computing an essential technology that was integral to their performance as future clinicians.
USA [210]	105	PDA	To assess both confort levels and self-described skill level before and after medical students class.	PDA class is beneficial, necessary, and should remain mandatory to faculty, students, and instructors.
USA [226]	227	PDA	To describe the prevalence and patterns of use of PDAs by nurse practitioner (NP) students and faculty, examine relationships between patterns of use of PDAs and demographic characteristics of NP students and faculty, and describe patterns of use of PDAs that support evidence-based practice (clinical scholarship).	PDAs may facilitate the application of evidence-based knowledge to practice.
Israel [253]	45	PDA	To determine residents' perception of the utility of PDAs and their influence on clinical practice at two teaching hospitals, one of	Palm owners in both groups responded that they used these devices to organize their record keeping and the most

			which subsidized resident purchase of a PDA.	frequently used programs were pharmacopoeias, medical reference and clinical calculators.
Australia [270]	76	PDA	To investigate whether PDAs would enhance students' pharmacological and clinical contextual knowledge and to identify issues associated with the use of PDAs in students' clinical experience.	Students using the PDAs demonstrated a moderate increase in their mean score, which was double the increase in the control group. Students found the PDAs easy to use and perceived their use as beneficial to their learning in the clinical area.
Canada [271]	36	PDA	To determine if the use of a PDA influences the students' preparedness for the safe administration of medications and enhances the students' self-efficacy.	There was a significant increase in self-efficacy in the groups with PDAs.
USA [272]	72	PDA	To evaluate the educational effectiveness of a PDA-based geriatric assessment tool.	Residents who used a PDA-based geriatric assessment tool demonstrated greater improvement in geriatric knowledge than peers who did not use this resource
USA [273]	122	PDA	To determine if a PDA-based smoking cessation counselling (SCC) tool can improve medical student smoking cessation counselling.	A PDA-based tool did not increase key SCC behaviors compared with a paper-based reminder. Students reported discomfort using the PDA with patients, lack of time,

				and lack of training as barriers to use of the tool.
USA [274]	30	iPod	To determine whether participation in educational conferences utilising iPod technology enhances both medical knowledge and accessibility to educational content among medical residents in training	iPod capture of conferences is a reasonable resource to help meet the educational goals of residents and residency programs.
